# N-Confused Metalloporphyrin-Based Electrocatalysts for Oxygen Reduction

**DOI:** 10.3390/molecules31111809

**Published:** 2026-05-25

**Authors:** You Luo, Zhuo Li, Jing Xie

**Affiliations:** Key Laboratory of Cluster Science of Ministry of Education, School of Chemistry and Chemical Engineering, Beijing Institute of Technology, Beijing 100081, China

**Keywords:** oxygen reduction reaction, first coordination sphere, reaction mechanism, electrocatalysis, architecture of catalysts, homogeneous and heterogeneous catalysis

## Abstract

Inspired by natural porphyrin-containing enzymatic active sites, metalloporphyrins have become important platforms for oxygen reduction reaction (ORR) catalysis because of their well-defined structures and tunable coordination environments. Recently, breaking the N_4_-coordination environment of cobalt porphyrins by inverting one pyrrolic unit to generate N_3_C_1_-site, i.e., N-confused porphyrin, has emerged as an effective strategy to promote their electro-catalyzing ORR capability. Herein, we reviewed recent progress in N-confused cobalt porphyrin in catalyzing ORR, with special emphasis on the influence of the catalyst’s architecture. We first summarized the general ORR mechanism on metalloporphyrins and the computational methods commonly used for mechanistic studies. Then, for comparison, the more common modification strategies like meso- and β-position substitution, axial coordination, and dinuclear design were reviewed for cobalt porphyrin-based catalysts. The main part reviewed the N-confused cobalt porphyrins with three different architectures, i.e., molecular, framework, and supported heterogeneous molecular form, highlighting their synthesis, characterization, electrocatalytic ORR behavior, and mechanistic interpretation from both experimental and theoretical perspectives. It summarizes the current understanding of why CoN_3_C_1_ systems outperform the original CoN_4_ porphyrin systems. The architecture of catalysts was found to affect the selectivity and mechanisms of ORR, along with the discussion of pH. The effects of N-confused strategy were compared to other modification strategies. Finally, we proposed possible directions for integrated catalyst design and mechanism studies.

## 1. Introduction

The reduction in dioxygen (O_2_) plays a vital role in both biological energy conversion and the development of artificial clean energy technologies. In nature, the oxygen reduction reaction (ORR) is essential to biological respiration. For example, cytochrome c oxidase (CcO) efficiently catalyzes the reduction of oxygen to water. This reduction process is coupled with transmembrane proton transfer, thereby driving the synthesis of adenosine triphosphate (ATP), which is the primary energy currency of living organisms. At the same time, ORR is an important half-cell reaction in advanced energy devices, including fuel cells and metal–air batteries. In these systems, oxygen functions as an electron or cation acceptor, and its reduction is coupled with the oxidation of fuels (H_2_, methanol, etc.), thereby enabling the efficient conversion of chemical energy into electrical energy. This mechanism underpins the generation of clean electricity for a broad range of applications, from portable electronic devices and electric vehicles to stationary residential power systems. To effectively convert chemical energy to electrical energy, an ideal ORR catalyst is expected to exhibit high rates, selectivity, and energy efficiency, as well as be cost-effective and environment friendly.

The ORR is a complex electrochemical process that involves the transfer of multiple electrons and protons. In protic solution, ORR occurs via two primary pathways, as represented below.

O_2_ + 2H^+^ + 2e^−^ → H_2_O_2_ (1)

O_2_ + 4H^+^ + 4e^−^ → 2H_2_O (2)

One pathway is the two proton/two electron (2H^+^/2e^−^) pathway that generates hydrogen peroxide (H_2_O_2_) as the product. H_2_O_2_ is an important chemical widely used in various industrial processes, such as chemical synthesis, wastewater treatment, and pulp bleaching. Hence, the production of H_2_O_2_ through the selective control of the ORR holds significant industrial application potential and substantial economic value. The other pathway is four proton/four electron (4H^+^/4e^−^) pathway that generates water as the product. This 4H^+^/4e^−^ pathway can maximize the energy output, so it is highly desirable in most energy applications such as fuel cell and metal–air batteries. However, H_2_O_2_ generated via competing pathways can be corrosive to the electric devices. From the perspective of both energy efficiency and operational safety, the formation of H_2_O_2_ is undesirable and should be avoided. In the 4H^+^/4e^−^ pathway, breaking the strong O–O bond, whose bond energy is ca. 498 kJ/mol, is an essential step and, concurrently, the most difficult step. Hence, reducing the barrier of O–O bond cleavage is one key factor of designing ORR catalyst towards H_2_O.

Considerable efforts have been dedicated to exploring new catalysts for the ORR [1,2,3,4]. Up to now, the most efficient materials for catalyzing the ORR are pure platinum and its alloys [5,6,7]. However, the high cost and limited geographic abundance of platinum group metals impede its commercial application and have driven intensive research toward economically viable alternatives, such as non-precious metal catalysts [8,9]. Among the potential ORR catalysts, metalloporphyrin-based materials have attracted considerable attention [10,11,12]. Such attention stems from their biomimetic structure, as well as their alignment with the widely embraced concept of single-atom catalysis in materials science [13,14,15,16]. In nature, cytochrome c oxidase (CcO) is the terminal enzyme in the mitochondrial respiratory chain, and it utilizes an iron porphyrin core structure at the heme a3-Cu_B_ site to reduce O_2_ to water [17,18,19,20]. Other metalloporphyrin examples include iron porphyrins (e.g., heme and cytochrome P450) [21], magnesium porphyrins (e.g., chlorophyl II) [22], and cobalt porphyrins (e.g., vitamin B12) [23].

**Figure 1:**
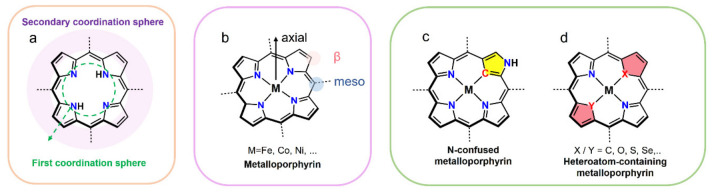
Structures of (**a**) porphyrin, (**b**) metalloporphyrin complexes, (**c**) N-confused metalloporphyrin complexes and (**d**) heteroatom-containing metalloporphyrin complexes.

As shown in Figure 1, porphyrin macrocycles consist of four pyrrole units connected by four methine bridges, forming a conjugated ring with 18 delocalized π electrons within an aromatic framework. This structural feature endows porphyrins with strong absorption in the visible light region. Upon deprotonation of the two N-H groups, dianionic porphyrin ligands are formed, which provide a rigid square-planar N_4_ coordination pocket that sterically accommodates most transition metal ions M. The resulting metalloporphyrin complexes have a planar M-N_4_ environment. In recent decades, the catalytic applications of metalloporphyrins have extended to a broad range of targets, encompassing the ORR, carbon dioxide reduction, as well as hydrogen and oxygen evolution reactions [17,24,25,26].

Metalloporphyrins possess a range of features that make them an ideal platform for designing novel ORR catalysts. The stable, well-defined, and planar structure of the molecular macrocycle allows for modifying functional groups at its *meso*- [27,28,29,30,31], β positions [32,33,34] or the adding of axial ligands [35,36,37], so as to regulate their physical and chemical properties and to carry out mechanistic investigations. In addition, the redox-active nature of porphyrin ligands allows them to participate in electron-transfer processes, making metalloporphyrins valuable for multi-electron catalytic reactions, such as ORR. Although metalloporphyrin-based molecular catalysts exhibit high ORR activity in homogeneous catalysis, their intrinsic semiconducting nature limits their utilization as heterogeneous electrocatalysis in the practical application of energy conversion processes in fuel cells and Zn-air batteries. Therefore, to transform homogeneous molecular catalysts into heterogeneous electrocatalysts, researchers tend to either immobilize the catalysts on a stable and conductive support or incorporate them into hierarchical frameworks [38,39,40].

A number of recent review articles have provided comprehensive coverage of utilizing metal porphyrin-based catalysts for ORR [24,40,41]. These reviews focused on traditional metalloporphyrin systems where the central metal is coordinated to four nitrogen atoms, displaying symmetric MN_4_ coordination. Such a highly symmetric, planar coordination structure, however, is not always beneficial for catalysis, because the uniform environment may limit differential control over substrate approach and intermediate stabilization [42]. Nonplanarity was found to endow porphyrins with functions not accessible to conventional planar analogs [43,44]. The electrostatic or steric asymmetries introduced by axial ligand, second-sphere substitution, or a second metalloporphyrin site can alter ORR selectivity and improve catalytic behavior [45,46,47]. Intriguingly, breaking the N_4_ symmetry can significantly boost catalytic performance [48,49,50,51,52,53]. Of note, this is a strategy widely used in graphene-based M-N-C-type single-atom catalyst design [54,55,56]. As for metalloporphyrin, the approaches to breaking the MN_4_ symmetry include creating N-confused porphyrins (NCPs) [57,58,59], where the inversion of one or two pyrrolic units gives unique N_3_C or N_2_C_2_ coordination (Figure 1c), or substituting N atoms by O/S atoms to create N_3_O, N_2_O_2_, N_3_S, N_2_S_2_ coordination (Figure 1d) [60,61]. Up to now, multiple N-confused and O/S-substituted metalloporphyrins have been synthesized with different metal centers, mainly 3d and 4d transition metals [26]. For N_3_C-coordination N-confused metalloporphyrin, a series of transition metals were reported to be synthesized, including Mn, Fe, Co, Ni, Cu, Mo, Rh, Pd, Ag, Re, Pt, Au, and Hg [62,63,64,65,66,67,68,69,70,71,72]. For N_2_C_2_ coordination, Cu and Ag have been reported [73,74]. For N_3_O, M can be Mn, Fe, Co, Ni, Cu, Zn, and Re [75,76,77,78]. For N_2_O_2_, only Ni has been reported [77]. For N_3_S, M can be Fe, Ni, Cu, Ru, Rh, Pd, Re, and Hg [75,79,80,81,82,83]. For N_2_S_2_, M can be Ru and Re [84,85].

**Figure 2:**
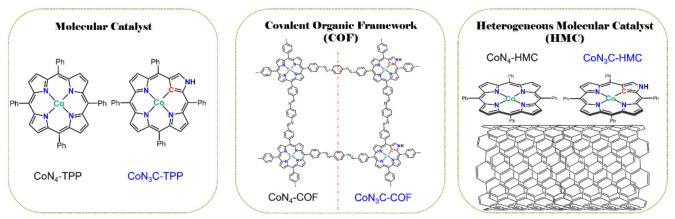
Metalloporphyrin and N-confused metalloporphyrin as molecular catalyst, incorporated in covalent organic framework and supported by nanotubes.

The most studied NCPs have a central metal of cobalt. This is largely attributed to the good performance of Co-porphyrins towards ORR as well as the feasibility of synthesizing Co-NCPs [49,50,86,87,88]. Intriguing, in the study of Co-NCP-based electrocatalysts, three distinct strategies have been employed to make molecular complexes heterogenized for electrocatalysis (Figure 2). Huang et al. synthesized metalloporphyrin molecular catalysts and used carbon black as a support in electrochemistry [49]. Shao et al. incorporated the porphyrinic units into highly ordered crystalline covalent organic frameworks (COFs) [50], and Liu et al. developed heterogeneous molecular catalysts by anchoring the macrocycles onto carbon nanotubes (CNTs) [52].

A comprehensive review of N-confused metalloporphyrins for ORR performance is lacking, especially regarding how the architecture or state of the catalysts influences the catalytic capability, and how the strategy of N-confusion compares to other modification strategies. To address this gap, this review focuses on the recent advances in N-confused cobalt porphyrin catalysts for ORR. This review is organized as follows. Section 2 introduces the general mechanisms of the oxygen reduction mechanism and the commonly used computational methods for mechanism studies. Section 3 briefly reviews the study on cobalt porphyrins in ORR catalysis and summarizes the influence of multiple modification strategies. Section 4 reviews the advances of N-confused cobalt porphyrins for ORR, with a focus on the difference in the architecture of the catalysts. A comparison between N-confused and other strategies mentioned in Section 3 will be presented. The review is closed up with a summary and outlook. By reviewing recent progress in first-coordination-sphere modification of metalloporphyrins, we aim to provide insights into the structure–activity relationships governing ORR catalysis and to offer guidance for the rational design of next-generation catalysts for sustainable energy conversion.

## 2. Mechanism of ORR on Metalloporphyrins

### 2.1. General Mechanism of ORR on Metalloporphyrins

**Figure 3:**
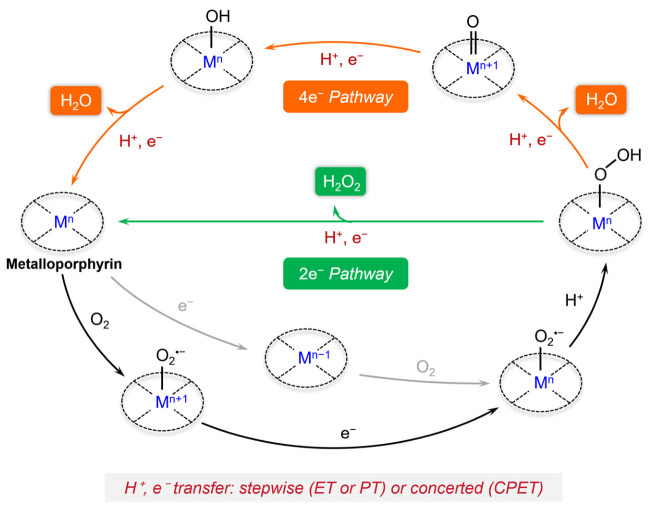
Possible reaction pathways for electrocatalytic O_2_ reduction by metalloporphyrins.

The performance of metalloporphyrin catalysts is fundamentally rooted in the molecular-level reaction pathway of ORR, making mechanistic understanding indispensable for interpreting and guiding catalyst design. In electrocatalysis experiments, electrons are transferred from the electrode to the catalytic system—sometimes via a conductor—which in turn reduces the target molecules. As shown in Figure 3, in homogeneous metalloporphyrin systems, the initial step of ORR is system-dependent, being either reduction in the catalyst or the coordination of O_2_ [89]. For example, in iron porphyrin systems, the catalyst is typically reduced to a low-valent state prior to O_2_ binding, after which O_2_ coordinates to form a metal–superoxo intermediate [90]. In contrast, in certain cobalt porphyrin systems, O_2_ can directly coordinate to the metal center before reduction, followed by electron transfer to generate a M-O_2_^•−^ species [91]. Both pathways ultimately converge to the formation of metal–superoxo intermediates as key species in the catalytic cycle. This is followed by the transfer of one proton and one electron, yielding a metal–hydroperoxo intermediate M-OOH. At this stage, the reaction bifurcates: if the O-O bond is cleaved upon further proton and electron transfer, the four-electron pathway proceeds to produce H_2_O via high-valent metal–oxo and metal–hydroxo intermediates; if the O-O bond remains intact, protonation releases H_2_O_2_ as the product, corresponding to the two-electron pathway. Therefore, the competition between O-O bond cleavage and hydroperoxo release fundamentally determines the selectivity of ORR in molecular metalloporphyrin systems. Notably, these proton- and electron-transfer steps can occur either in a stepwise manner (PT then ET, or ET then PT) or via a concerted proton-coupled electron transfer (CPET), depending on the electronic structure of the catalyst, the electrolyte, and the applied potential, which collectively influence the coupling [85] of electron and proton transfer [92].

Beyond the general mechanistic framework, many factors can influence the exact ORR mechanism and selectivity. Some factors include the following: the type of central metal, the reaction environment like pH, the nature of support materials, and the structural modification of metalloporphyrins on the first/second coordination sphere. They affect the electronic structure of catalysts, the adsorption of O_2_, electron and/or proton-transfer behavior, intermediate stability, etc. [93]. The ORR mechanism on metalloporphyrins is governed by the interplay between metal-centered redox chemistry, ligand electronic effects, proton-coupled electron-transfer processes and electrochemical reaction conditions. Catalytic performance is largely determined by the ability to balance the stabilization and transformation of oxygenated intermediates, particularly hydroperoxo species. A key challenge lies in elucidating the intrinsic relationship between these influencing factors and the regulation of the reaction mechanism. A complete mechanistic understanding of ORR relies on the combined use of theoretical and experimental approaches.

### 2.2. Computational Studies on ORR Mechanisms

Density functional theory (DFT) calculations are widely employed to model intermediates, evaluate reaction free energies, and identify potential-determining steps within the catalytic cycle. Electronic structure analyses, such as charge distribution and orbital interactions, further provide insight into how ligand modifications influence catalytic behavior [94]. In theoretical studies, the mechanistic analysis of ORR catalyzed by metalloporphyrins is almost entirely based on density functional theory (DFT). Existing work mainly follows two modeling strategies, namely cluster-based DFT calculations and periodic DFT calculations.

For molecular metalloporphyrin complexes that act as homogeneous catalysts, mechanistic studies usually employ cluster models and are commonly carried out with quantum chemistry packages such as Gaussian under implicit or explicit solvation conditions. In such systems, electron transfer and proton transfer can often be treated as separate steps. As a result, in addition to common oxygen-containing intermediates such as the *O_2_, *OOH, *O, and *OH, species with different reduction states and different protonation states also need to be considered. For these candidate intermediates, geometry optimizations and vibrational frequency calculations are typically performed to determine whether they correspond to stable minima or transition states, while the relative energies of different spin states are compared to identify the ground-state structure. On this basis, Gibbs free energy comparisons are then used to identify the true active species and the preferred reaction pathway. In addition, reduction potentials and p*K*_a_ values of key species are often calculated to clarify the sequence of electron-transfer and proton-transfer steps and to determine whether the reaction is more likely to proceed through a stepwise ET/PT pathway or may involve a concerted proton-coupled electron-transfer process.

For heterogeneous metalloporphyrin catalysts, mechanistic studies are usually carried out using periodic DFT calculations, most commonly with plane-wave packages such as VASP. Typical computational settings include the PBE functional, PAW pseudopotentials, and D3 dispersion correction. Unlike homogeneous molecular systems, heterogeneous ORR studies usually treat concerted proton–electron transfer as a unified process. Accordingly, electron transfer and proton transfer are generally not discussed separately. Instead, reaction free energy diagrams are constructed directly around O_2_ adsorption and the key oxygen-containing intermediates *OOH, *O, and *OH. In other words, the central task in such calculations is to determine the adsorption configuration of O_2_ at the active site, calculate the adsorption free energies of *OOH, *O, and *OH, and then compare the free energy changes in the elementary steps. This allows identification of the true active site, the preferred reaction pathway, the potential-determining step, and the theoretical overpotential. Beyond free energy diagrams, these studies are also often combined with adsorption-energy analysis, overpotential analysis, comparisons between two-electron and four-electron selectivity, and stability analyses based on metal binding energies, aggregation tendencies, and dissolution potentials, in order to explain the activity, selectivity, and stability of the catalyst under electrochemical conditions. For the two-electron ORR pathway, it is usually sufficient to further compare whether *OOH continues to be reduced to *O or desorbs directly to form a peroxide species. Therefore, the stability of *OOH is often the key factor that distinguishes the two-electron pathway from the four-electron pathway.

From a thermodynamic perspective, mechanistic calculations for heterogeneous ORR usually employ the computational hydrogen electrode (CHE) model to describe the energy change associated with the addition of one proton and one electron, which is the effective addition of one hydrogen atom. Within this framework, the chemical potential of a proton–electron pair is usually represented by 1/2 *G*(H_2_), and the reaction free energy of each elementary step is generally written as Δ*G* = Δ*E* + ΔZPE − *T*Δ*S* + Δ*G*_U_ + Δ*G*_pH_. Here, Δ*E* is the DFT total-energy difference, ΔZPE and *T*Δ*S* correspond to the zero-point energy and entropy corrections, respectively, Δ*G*_U_ = −ne*U* is used to account for the effect of the applied potential, and ΔG_pH_ describes the correction arising from the solution pH. In practical calculations, the adsorption free energies of *OOH, *O, and *OH are usually obtained first, and the reaction free energies of the elementary steps are then derived from the relative free energy differences among these intermediates, which further allows evaluation of the theoretical overpotential. Under acidic conditions, the overall reaction is usually written as O_2_ + 4H^+^ + 4e^−^ → 2H_2_O, and the elementary steps can be described as the sequential uptake of H^+^ and e^−^ by the adsorbed intermediates. Under alkaline conditions, the reaction is usually rewritten as O_2_ + 2H_2_O + 4e^−^ → 4OH^−^, where the proton is no longer treated as free H^+^; instead, H_2_O is taken as the proton source and OH^−^ is written as the product in the corresponding elementary steps. Thus, although both acidic and alkaline systems can be analyzed within the CHE framework, the reaction expressions, proton source, and the treatment of the pH correction term still need to be considered separately.

## 3. Cobalt Porphyrins for ORR

Cobalt tetraphenylporphyrins (CoTPP, **1**) is the pristine model for the study and optimization of ORR performance. Regulation strategies include *meso*- or β-position modification, axial coordination tuning, and the construction of two neighboring Co sites. According to the oxo-wall rule [95], late transition metals (including Co) in monometallic porphyrins tend to reduce O_2_ to H_2_O_2_, because the metal–oxo intermediate, a necessary intermediate for the reduction to H_2_O, is unstable [96,97]. In fact, the ORR products of mononuclear cobalt porphyrins include both H_2_O and H_2_O_2_ [17,89]. Hence, the selective reduction is a key goal and a major challenge. In this section, we will briefly review examples of cobalt porphyrin compounds that apply the above four modification strategies, focusing on the changes in ORR activity and selectivity, as well as the underlying mechanisms. These examples are summarized in Table 1.

### 3.1. Effects of Meso-Position Substitution

**Figure 4:**
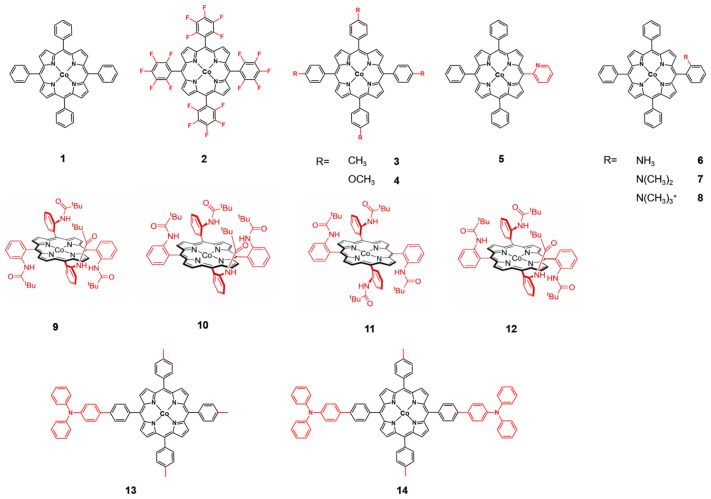
Molecular structures of cobalt porphyrins **1**–**14**.

Depending on the type of functional groups introduced at the *meso*-positions, the mechanism to influence ORR activity and/or selectivity varies (Figure 4). In comparison to CoTPP (**1**), introducing strong electron-withdrawing group (like F) to the *meso*-phenyl ring to form tetra-pentafluorophenyl cobalt porphyrin (CoTFPP, **2**) lowers the electron density on Co and reduces the ORR activity under basic condition, i.e., the half-wave potential decreases from 0.81 V (**1**) to 0.76 V (**2**) and the Tafel slope increases from 36.9 to 43.5 mV dec^−1^ [98]. In contrast, introducing electron-donating methyl (**3**) and methoxy (**4**) groups to the *meso*-phenyl ring [99] helped stabilize partially reduced intermediates near the Co center, allowing them to accept additional electrons and undergo further reduction to H_2_O, hence improving both selectivity and activity. CoTPPy (**5**) has a 2-pyridyl group at one *meso*-position; under acidic condition and an applied potential of 0.4 V, the electron-transfer number was reported to be 3.51, in comparison that of 2.6 for CoTPP [100]. This 4H^+^/4e^−^ pathway selectivity was attributed to a proton-relay effect, for the microenvironment around the Co center was altered to promote proton transfer. This view is further supported by the work of Co hangman porphyrins [105]. Substituting one H on one of the meso-phenyl rings by NH_2_, NMe_2_, and NMe_3_^+^, and forming CoTPPNH_2_ (**6**), CoTPPNMe_2_ (**7**), and CoTPPNMe_3_^+^ (**8**) [45], changes the selectivity under different pH (0, 4, and 7). Among them, complex **8** has a greater preference towards the 4e^−^ reduction pathway as the electron-transfer number is the largest (3.2–3.8). Here, the electrostatic effect of cationic functional group was proposed to play a role during proton transfer, thus influencing the selectivity. When the functional group is bulk, the spatial arrangement and steric effects play a role. Complexes **9**, **10**, **11**, **12** are cobalt porphyrin atropisomers bearing four ortho-amido groups at *meso*-position. Although they have similar intrinsic onset activity, i.e., half-wave potentials are 0.72–0.73 V, the αααα isomer (with n of 3.75) favors the 4e^−^ pathway. This is because the highly symmetric αααα isomer forms a more favorable pocket on one side for O_2_ access and binding, resulting in subsequent O_2_ activation and O-O cleavage at the Co center. The effect of spatial arrangement was also reflected on the comparison between the asymmetric aBz-TCoP (**13**) and the symmetric catalyst Bz-2TCoP (**14**) [101], where complex **14** has higher n values (3.6/3.5) under acidic/alkaline conditions in relative to **13**. Overall, *meso*-position modification can regulate ORR activity and selectivity by tuning the electronic structure, proton-transfer environment, and steric pocket around the Co center.

### 3.2. Effects of β-Position Substitution

**Figure 5:**
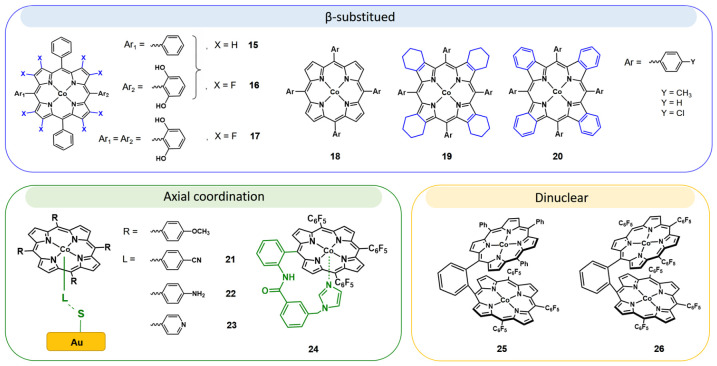
Molecular structures of cobalt porphyrins **15**–**26**.

In terms of β-position modification (Figure 5), introducing electron-withdrawing F groups to all the eight β-positions of the CoTPP shifts the reduction potential to be more positive—from −1.36 V for CoTP(OH)_2_ (**15**) to −0.97 V for CoTPF_8_(OH)_2_ (**16**), and −1.01 V for CoTPF_8_(OH)_4_ (**17**) [102]. Because β-F substitution lowers the energy level of the porphyrin ligand π* orbitals, the system becomes more likely to accept electrons, i.e., to be reduced. In addition, the β-fluorinated systems showed higher selectivity toward H_2_O, and DFT calculations revealed that β-F substitution strengthened the binding of H_2_O_2_-related intermediates, making their premature release less favorable and thus promoting their further reduction to H_2_O. The other work extended the conjugation of Co-TPP by substituting benzyl rings at β-positions [33]. The resulting benzo fusion complexes **20** displayed increased favor for 4e^−^ reduction (i.e., greater n values), attributed to the strengthened intermolecular π–π interactions. Overall, β-position modification mainly affects ORR by tuning the electronic structure and π-conjugation of the porphyrin ligand, and it can promote the 4e^−^ pathway by strengthening intermediate binding or intermolecular interactions.

### 3.3. Effects of Axial Coordination

Tuning axial coordination directly affects the electronic structure of the Co center, hence impacting O_2_ binding, activation, and the O-O bond cleavage process. For example, [tetrakis(4-methoxyphenyl) porphyrin]cobalt, denoted TMPPCo, was assembled on thiol-modified Au electrodes and three electron-donating ligands were introduced as axial ligands, including 4-mercaptopyridine (MPy, **21**), 4-aminothiolphenol (APT, **22**) and 4-mercaptobenzonitrile (MBN, **23**) [36]. All three systems proceed with the 2e^−^ pathway. The ORR onset potential shifted to be more positive: 0.53 V (**21**), 0.48 V (**22**), and 0.45 V (**23**) vs. RHE. The catalytic activity order follows the coordination ability order of the ligands: MPy > APT > MBN. DFT calculations suggested that axial ligands increase electron donation to the Co center through a push effect, decreases its positive charge, and enhances back-donation to O_2_, thus facilitating O_2_ adsorption and O-O bond activation. In another example [89], a Co porphyrin **24** with an appended imidazole group for Co axial binding was synthesized. Similarly, O_2_ binding and activation at the Co site was significantly improved through an electron-pushing effect. Complex **24** is more effective than its structural analog parent compound **2**, which lacks the appended imidazole group, in terms of mediating ORR. Moreover, since the axial imidazole group compactly incorporated into the molecular framework, it affects the conversion of the O_2_ adduct and subsequent oxygen-containing intermediates, hence affecting the 2e^−^/4e^−^ selectivity. After loading both molecules onto CNTs and testing them for ORR in 1.0 M KOH, **24** exhibited a half-wave potential of 0.83 V and an electron-transfer number of 3.29, in comparison to 0.70 V and the n value of 2.78 for complex **2**. The formation of H_2_O was found to through a direct 4e^−^ reduction pathway. As a practical demonstration, the Zn–air battery assembled with **24** as the air electrode catalyst displayed comparable performance to the battery using Pt/C + Ir/C. Specifically, **24**-based Zn–air battery delivered a smaller charge–discharge voltage gap (0.88 V vs. 0.91 V), a higher specific capacity (785 vs. 735 mA h g^−1^), and a higher peak power density (120 vs. 75 mW cm^−2^) than the battery based on Pt/C + Ir/C, while also showing high cycling durability for over 60 h. In brief, axial coordination regulates ORR mainly through an electron-pushing effect on the Co center, which enhances O_2_ adsorption and activation, promotes O-O bond activation, and further influences the 2e^−^/4e^−^ pathway depending on the axial ligand structure.

### 3.4. Effects of Dinuclear

The idea of constructing binuclear porphyrin catalysts also originates from biological enzyme systems. In multi-electron redox enzymes, multiple metal centers often work together in substrate binding and the subsequent redox process [37]. Therefore, researchers have constructed binuclear cobalt porphyrin structures in which two Co sites are placed in close proximity. Binuclear Co porphyrin molecules can selectively catalyze the 4e^−^ ORR by forming a Co-O_2_-Co intermediate.

Cao group synthesized asymmetric Pacman dinuclear cobalt porphyrin complex **25**, featuring triphenylporphyrin (TPP) and tri(pentafluorophenyl)-porphyrin (TPFP) ligands, outperforming its symmetric analog **26**, (Co-TPFP)_2_. Both binuclear cobalt porphyrins catalyze the 4e^−^ ORR more efficiently and with higher selectivity than the corresponding mononuclear Co^II^ porphyrins, CoTPP (**1**) and CoTPFP (**2**). **25** exhibits a higher catalytic current and a lower overpotential compared to **26** in 0.5 M H_2_SO_4_. According to the DFT studies by Liao group, the enhanced efficiency of complex **25** stems from its asymmetric structure: the Co-TPP unit serves as the primary site for O_2_ binding and reduction, while the positively charged Co-TPFP moiety acts as a Lewis acid, promoting O_2_ activation. Moreover, Bronsted acid H_2_SO_4_ facilitates O-O cleavage via proton donation and hydrogen bonding, lowering the barrier for this critical step and stabilizing intermediates. It can be seen that asymmetric binuclear metal catalysts play a crucial role in the electrocatalytic 4e^−^ ORR process. Nocera et al. found that installing trans aryl groups on binuclear Co-porphyrins reduces the selectivity for the direct reduction of O_2_ to H_2_O [106]. Cook et al. synthesized three types of face-to-face binuclear cobalt porphyrin systems with different distances via self-assembly. They found a shorter bridging ligand, which involves two Ru centers and improves 4e^−^ selectivity and the overall reaction rate constants, suggesting that the spatial arrangement of the two Co sites affects the cooperative effect between them during ORR [46]. To sum up, binuclear cobalt porphyrins promote 4e^−^ ORR by creating cooperative dual-Co sites, where the relative distance, orientation, and electronic asymmetry between the two Co centers determine O_2_ binding, O-O bond cleavage, and the efficiency of the reduction to H_2_O.

From the above analysis, it can be seen that meso-substituents and β-substituents (i.e., electron-withdrawing and electron-donating effects), axial ligands (i.e., electron push effects), the secondary coordination sphere structure (i.e., proton relay), and the number of metal centers all have a significant impact on the ORR of cobalt porphyrins. When these effects are present individually or simultaneously in the porphyrin, their influences on ORR activity, selectivity, and stability differ. It is worth mentioning that carbonaceous support/conductive additive also affects ORR performance. As shown in Table 1, multiple types of supports including CNT, GCE, EPG, and CB were used. Such carbon materials can substantially affect the local interfacial microenvironment surrounding the active sites, including conductivity, oxygen mass transport capability, hydrophobicity/hydrophilicity, and electronic coupling with the catalytic centers. The effects of supports on the ORR performance and selectivity of CoN_4_-TPP and CoN_3_C-TPP will be discussed in the next section.

## 4. N-Confused Cobalt Porphyrins for ORR

The above studies on cobalt porphyrin-catalyzed ORR retained the coordination environment of CoN_4_. In this section, we will discuss how modifying the primary coordination sphere affects the performance of cobalt porphyrins in ORR. This discussion is primarily based on three recent works from different groups. All these works used CoTPP as the pristine model and the reference system, and synthesized N-confused Co porphyrin derivatives with an N_3_C_1_ coordination environment. All three studies demonstrated that breaking the symmetric N_4_ structure and introducing a carbon atom to form an asymmetric N_3_C_1_ coordination environment leads to a catalyst with a higher ORR activity (i.e., lower ORR overpotential) and changes ORR selectivity (Table 2).

Interestingly, the architectures of these catalysts differ: Huang et al. synthesized a CoN_3_C_1_-TPP molecular catalyst (denoted as CoN_3_C_1_-TPP molecular system), Shao et al. group-embedded the CoN_3_C_1_ porphyrin unit into a covalent organic framework (denoted as CoN_3_C_1_-COF system), and Liu et al. non-covalently anchored CoN_3_C_1_-TPP onto the surface of multi-walled carbon nanotubes (MWCNTs) to construct a heterogeneous molecular catalyst (denoted as CoN_3_C_1_-HMC). Since the three works show good consistency in content and logic, we will make one-to-one comparisons in the following aspects: DFT-based screening and prediction, synthesis and characterization of N-confused porphyrin catalysts, electrocatalytic ORR performance, and mechanism analysis. Finally, a discussion between N-confused strategy and other modification methods in Section 3 will be presented.

### 4.1. DFT-Based Screening and Prediction

The first part of the three papers is screening by DFT calculations to predict the proper central metal for the N-confused metalloporphyrin-based catalysts. Although the computational models and methods are different, the calculations reached the same conclusion that N-confused porphyrin systems with Co as the central metal show improved ORR catalytic performance compared to MN_4_ systems, making them the most promising electrocatalysts.

**Figure 6:**
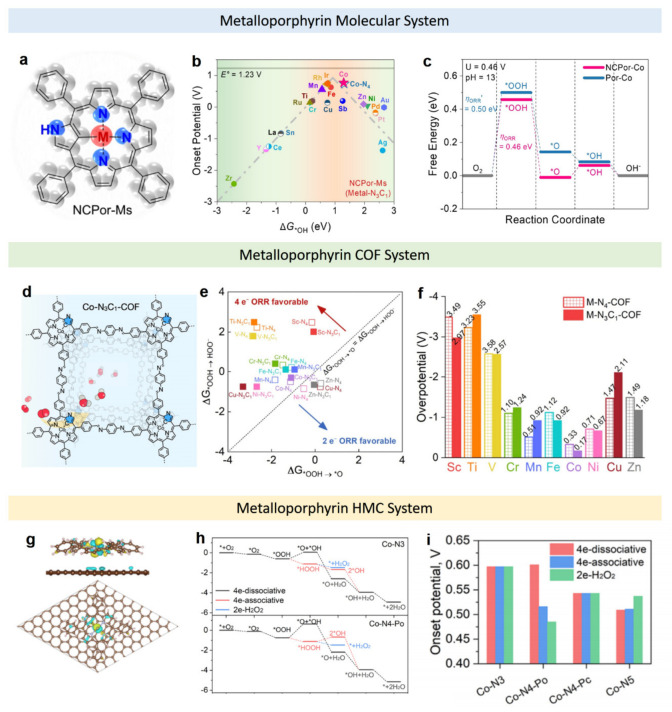
DFT-based screening and mechanistic analysis of N-confused metalloporphyrin ORR catalysts in molecular (**a**–**c**), COF (**d**–**f**), and HMC systems (**g**–**i**). (**a**) Molecular structure of M-N_3_C_1_-TPP. (**b**) Volcano plot of calculated onset potential versus ΔG_*OH_ for M-N_3_C_1_-TPP with different metal centers. (**c**) ORR free energy profiles of CoN_3_C_1_-TPP and CoN_4_-TPP. (**d**) Structural model of CoN_3_C_1_-COF. (**e**) Selectivity descriptor based on the competition between *OOH further reduction and HOO^−^ release in M-N_4_-COF and M-N_3_C_1_-COF systems. (**f**) Calculated overpotentials of M-N_4_-COFs and M-N_3_C_1_-COFs with different metal centers. (**g**) Structural model of CoN_3_C_1_-HMC supported on graphene. (**h**) Free energy profiles of 2e-H_2_O_2_, 4e-associative, and 4e-dissociative ORR pathways on CoN_3_C_1_-HMC and CoN_4_-HMC. (**i**) Calculated onset potentials of different CoN_x_-HMCs for 2e-H_2_O_2_, 4e-associative, and 4e-dissociative pathways. Adapted from reference [49,50,52].

In terms of the simulation model, Huang et al. constructed the molecular cluster model (Figure 6a), Shao et al. constructed the single-layer periodic model of the COFs (Figure 6d), and Liu et al. constructed a periodic model with metalloporphyrin molecules on top of single-layer graphene (Figure 6g). The employed functional is PBE and the software is VASP. All the calculations assumed PCET and no transition states were considered.

Huang et al. designed 21 types of M-N_3_C_1_ molecular systems with group 3d, 4d, and 5d transition metals (Figure 6b). To evaluate the activity of the M-N_3_C_1_-TPP and CoN_4_-TPP, their calculated onset potentials were plotted against their adsorption free energies of *OH (∆G_*OH_) and *OOH (∆G_*OOH_), and displayed volcano curves (Figure 6b). According to the Sabatier principle, the best catalysts are located at the vertex of the volcano plots, so the M-N_3_C_1_-TPP active sites with M equals Co, Mn, Fe, Ir, and Rh are predicted to be promising ORR catalysts. The CoN_3_C_1_ site exhibits the highest ORR activity with *E*_onset_ of 0.77 V, and this is more positive than the corresponding CoN_4_ sites, where *E*_onset_ equals 0.73 V. The authors also computed a free energy profile encompassing the energetics of key intermediates *OOH, *O, and *OH, and the resulting overpotential *η*^ORR^ is 0.46 V for CoN_3_C_1_-COFs and 0.50 V for CoN_4_-COFs, which also suggests the N_3_C_1_ system has higher activity (Figure 6c). Of note, although the catalysts are molecular clusters in this work, the authors used VASP for calculation, which is usually used for the periodic system. We think the reason for this choice is to obtain *E*_onset_.

Shao et al. designed 10 types of M-N_3_C_1_-COFs and M-N_4_-COFs by varying the central metal from Sc to Zn, and computed the energetics of key intermediates *OOH, *O, and *OH. Among the 20 COFs, Co-based catalysts were predicted to have the lowest theoretical overpotential *η*^ORR^, 0.17 V for CoN_3_C_1_-TPP and 0.33 V for CoN_4_-COFs (Figure 6f). By comparing the Gibbs free energy changes (ΔG_*OOH→*O_) of *O formation against Gibbs free energy changes (ΔG_*OOH→HOO−_) of HOO^−^ formation, CoN_3_C_1_-COF was predicted to have a stronger preference for 4-electron reduction to H_2_O than the parent CoN_4_-COF (Figure 6e).

Liu et al. designed four types of organic molecules with cobalt center that are placed on a single layer of graphene sheet, where the parent organic molecules are N-confused porphyrin (CoN_3_C_1_-HMC), porphyrin (CoN_4_-HMC), phthalocyanine (CoN_4_), and texaphyrin (CoN_5_). To limit the scope of this review, we focus on the first two porphyrin systems. The binding energy difference between the *O_2_ and *HOOH intermediates (Δ*E* = E_*O2_ − E_*HOOH_) was used to qualitatively predict the H_2_O_2_ selectivity, and a close-to-zero value indicates high selectivity. The Δ*E* values for CoN_3_C_1_-HMC and CoN_4_-HMC are −0.22 eV and −0.41 eV, respectively, so the authors concluded that CoN_3_C_1_-HMC has a higher H_2_O_2_ selectivity. Then, ORR free energy profiles were computed for three pathways, i.e., 2e-H_2_O_2_, 4e-associative, and 4e-dissociative (Figure 6h). The resulting onset potentials *η*^ORR^ for the three pathways are all 0.597 CoN_3_C_1_, and are 0.485, 0.516, and 0.601 for CoN_4_, respectively (Figure 6i). Overall, *η*^ORR^ are more positive (meaning higher ORR activity) for CoN_3_C_1_-HMC, consistent with the other two works. We point out that the dissociative pathway, which involves *HOOH → 2*OH and the *OOH → *O + *OH step, are not common in the mononuclear metalloporphyrin homogeneous system; they are more common in bimetallic [94] and multimetallic systems, or heterogeneous catalysts.

### 4.2. Synthesis and Characterization of N-Confused Porphyrin Catalysts

Since all DFT calculations predicted that the N-confused cobalt porphyrin systems outperform the corresponding cobalt porphyrin-based catalysts, three experimental groups synthesized them with different architectures. Huang et al. directly synthesized the discrete M-N_3_C_1_-TPP molecules, where M = Mn, Co, Ni, Cu, Pd, and Ag, by the reaction between pyrrole and benzaldehyde, followed by metalation. Then, the molecules were physically mixed with conductive carbon black in an ethanol solution containing Nafion. The resulting homogeneous ink was uniformly drop-casted onto the electrode surface for electrocatalytic testing. Shao et al. constructed the COFs via imine condensation, where the formyl-functionalized cobalt N_3_C_1_-porphyrin monomer was reacted with p-phenylenediamine, affording the highly crystalline and porous 2D COF material. Liu et al. synthesized the CoN_3_C_1_-TPP molecules; then the molecules were firmly anchored onto the deep purified commercial multi-walled carbon nanotubes (MWCNTs) which were surface-driven by strong van der Waals interactions (specifically, π-π stacking). This strategy successfully forms heterogeneous molecular catalysts (HMCs) that combine the excellent conductivity of the carbon support with the well-defined asymmetric active sites of the molecular catalysts. DFT calculations further confirmed that the van der Waals interactions ensure the stability of the coupling, as the calculated binding energies between CoN_4_/CoN_3_C_1_-TPP and the graphene (that models the CNTs) are about −2.0 eV.

The three studies primarily utilized X-ray Absorption Spectroscopy (XAS), especially Extended X-ray Absorption Fine Structure (EXAFS) quantitative fitting and X-ray Absorption Near-Edge Structure (XANES), alongside other spectroscopic techniques to explicitly verify the asymmetric CoN_3_C_1_ coordination environment.

In the molecular catalyst work by Huang et al., the EXAFS spectra exhibited prominent first-shell peaks corresponding to Co-N and Co-C bonds. Through quantitative fitting, the coordination number (CN) was determined to be approximately 3.0 for the Co-N shell and 1.0 for the Co-C shell, providing direct structural evidence for the CoN_3_C_1_-TPP coordination. In addition, near-edge X-ray absorption fine structure (NEXAFS) at both the C K-edge and N K-edge displayed specific dipole transitions (such as transitions to π^∗^ (Co-N/C) orbitals), which further confirmed the presence of Co-N and Co-C bonds within the molecule.

**Figure 7:**
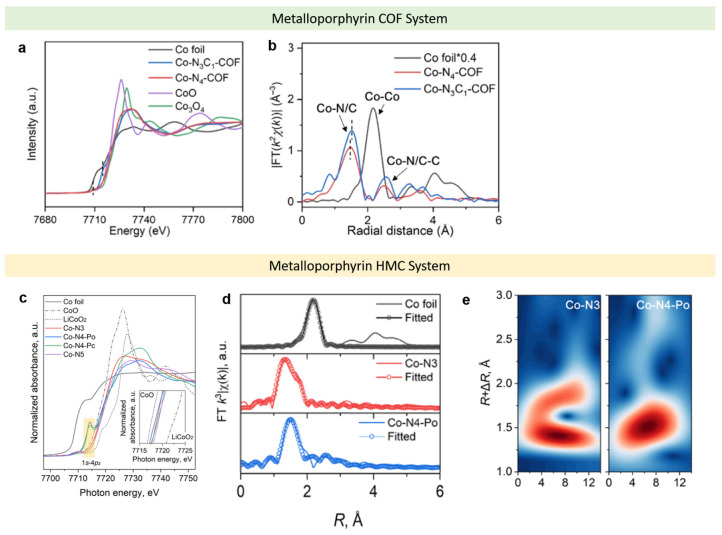
CoN_4_-COF and CoN_3_C_1_-COF system: (**a**) Co K-edge XANES spectra and (**b**) FT-EXAFS spectra. CoN_4_-HMC and CoN_3_C_1_-HMC system, the * in “Co foil*0.4” indicates that the intensity of the Co foil curve was multiplied by 0.4 for comparison, (**c**) Co K-edge XANES spectra, (**d**) EXAFS fitting results, (**e**) WT-EXAFS contour plots. Adapted from reference [50,52].

In the CoN_3_C_1_-COF work by Shao et al., the XANES spectra showed the disappearance of the characteristic 7709 eV fingerprint peak, which typically represents a symmetrical square-planar CoN_4_-TPP coordination. Instead, a new pre-edge peak emerged at 7716 eV (corresponding to a dipole-allowed 1s → 4p transition), confirming the highly distorted, asymmetrical coordination environment (Figure 7a). In addition, in the Fourier-transformed EXAFS spectra, the main peak for CoN_3_C_1_-COF shifted to 1.52 Å, noticeably different from the 1.46 Å peak of the symmetric CoN_4_-COF. This shift was attributed to the simultaneous formation of Co-N and Co-C bonds (Figure 7b). The quantitative fitting results confirmed a Co-N coordination number of 2.6 ± 0.3 and a Co-C coordination number of 0.9 ± 0.1.

In the CoN_3_C_1_-HMC work by Liu et al., the first-shell EXAFS fitting revealed a combination of paths: a three-fold Co-N path with a compressed bond length of ~1.916 Å, and a one-fold Co-C path with a bond length of ~2.106 Å (Figure 7d). Wavelet-transformed EXAFS (WT-EXAFS) contour plots also provided visual support for these distinct backscattering paths (Figure 7e). In addition, in the XANES Pre-edge Analysis, the asymmetrical CoN_3_C_1_-HMC catalyst exhibited a distinct pre-edge peak at ~7708.8 eV, which is ascribed to a parity-forbidden quadrupole 1s → 3d transition indicative of a highly distorted coordination geometry. This starkly contrasts with the strong pre-edge peak at ~7717 eV observed for planar D_4h_ symmetric CoN_4_-HMC catalysts (Figure 7c). Moreover, N K-edge electron energy loss spectroscopy (EELS) and N 1s XPS were used. Both techniques detected specific signals (a shoulder peak at ~399.2 eV in EELS and a peak at 400.4 eV in XPS) assigned to the unbounded pyrrolic “-NH” group, which is a unique structural signature of the N-confused porphyrin ring. We note that the assignment of the XANES peaks is not consistent in the COF and HMC systems. We believe that it is necessary to unify this phenomenon experimentally in the future, probably in combination with theoretical calculations.

It is worth mentioning that the stability of these asymmetric coordination structures in the harsh environment of electrocatalysis is critical. All these three articles directly address this issue and, through different ex situ or *operando* characterization techniques, consistently demonstrate that the configuration remains stable during the electrocatalytic ORR process, without undergoing irreversible structural collapse or reconstruction.

### 4.3. Electrochemical ORR Performance

The electrocatalysis ORR performance of these three N-confused cobalt porphyrin systems was evaluated using conventional three-electrode methods, including RRDE-LSV and RDE-LSV. A detail comparison is present in Table 2. Huang et al. and Shao et al. both carried out their measurements in O_2_-saturated 0.1 M KOH solution, and Liu et al. used an RRDE setup in acidic, neutral, and alkaline (0.1 M KOH) electrolytes to examine the pH-dependent behavior of the heterogeneous molecular catalysts (HMCs). Since 0.1 M KOH solution was used in these three works, a direct comparison between their ORR performances is possible. Of note, different conductive carbon materials were employed, including carbon black in Huang’s work, Ketjen Black in Shao’s work, and MWCNTs in Liu’s work. Nevertheless, all three studies found that changing CoN_4_ to asymmetric CoN_3_C_1_ coordination environment enhances the intrinsic ORR activity of the cobalt site, as reflected by more positive *E*_onset_ or *E*_1/2_ potentials and lower theoretical overpotentials *η*^ORR^.

As shown in Table 2, under 0.1 M KOH condition, the *E*_onset_ of CoN_4_/CoN_3_C_1_ systems are 0.77/0.83 V for molecular catalysts, 0.675/0.794 V for COFs, and 0.60/0.78 V for HMCs. The corresponding *E*_1/2_ of CoN_4_/CoN_3_C_1_ systems are 0.84/0.95 V, 0.82/0.89 V, 0.85/0.93 V, respectively. In comparison, changing the coordination from CoN_4_ to CoN_3_C_1_ shifts the *E*_onset_ towards the positive by 0.06–0.18 V, and the *E*_1/2_ by 0.07–0.11 V. In addition, the kinetic current density of CoN_3_C_1_-COF reached 72.1 mA cm^−2^, approximately 1.8 times that of CoN_4_-COF (0.32 V). When considering solely the absolute value of *E*_onset_ or *E*_1/2_ potentials, the CoN_3_C_1_ molecular catalysts appear to be the most effective ORR catalysts, exhibiting the most positive *E*_onset_ (0.95 V) and *E*_1/2_ (0.83 V). This is comparable to and even better than the Pt/C benchmark, with *E*_onset_ ≈ 0.92 V and *E*_1/2_ ≈ 0.81 V, as estimated from Figure 2b in the original article by Huang et al. [49].

The CoN_3_C_1_-Por molecular catalyst was further used as the air cathode of the rechargeable Zn–air battery, in which 6 M KOH with 0.1 M ZnCl_2_ was used as the electrolyte, and a polished Zn plate was used as the anode. Huang et al. reported that the maximum power density of the CoN_3_C_1_ molecular catalyst-based battery reaches 120.4 mWcm^−2^ at a current density of 207.1 mAcm^−2^. This is higher than those of the CoN4-Por molecular catalyst (66.5 mWcm^−2^ at 136.9 mAcm^−2^) and commercial 20% Pt/C (111.1 mWcm^−2^ at 169.7 mAcm^−2^). This battery shows no obvious voltage change after 70 h of charge–discharge cycles, revealing the high stability of CoN_3_C_1_-Por molecular catalyst in Zn–air batteries. We have the opinion that the HMC architecture would be more suitable for practical high current density Zn–air battery and fuel cell applications, albeit the existing literature did not perform such testing. This is because they tend to provide a more balanced integration of catalytic activity, electron transport, mass transport, and structural robustness.

In terms of selectivity under the 0.1 M KOH condition, all systems prefer the 4e^−^ pathway to 2e^−^ pathway; their H_2_O_2_ yields are smaller than 30%. The molecular catalysts and COFs, proceeding from a near-four-electron pathway, have better H_2_O selectivity than the HMCs. Changing the coordination from CoN_4_ to CoN_3_C_1_ affects the selectivity, but the trend is different among the three systems. The H_2_O selectivity was enhanced in molecular catalysts (slightly, n increases by 0.05–0.1) and COFs (greatly, n increases by 0.6); however, it was inhibited in HMCs (n decreases by 0.16). This illustrates that the support has a significant role in affecting the selectivity or ORR.

It would be interesting to understand why N-confusion increases 4e^−^ selectivity in molecular and COF systems but decreases it in CNT-supported HMCs. We speculate that different carbon supports exhibit distinct interfacial interactions and charge transfer behaviors with the catalytic sites; they may modulate the electronic structure of the active centers and consequently alter the adsorption strength of ORR intermediates, ultimately affecting both catalytic activity and reaction pathways. In particular, the intrinsic curvature and extended π-conjugated structure of MWCNTs may further influence the local electronic environment and interfacial electron delocalization of the supported molecular catalysts, thereby contributing to variations in ORR selectivity and performance [107,108,109]. Further studies are required to elucidate the exact role of the different supports.

The effect of pH on selectivity can be obtained from the tests on HMCs. In general, for the HMCs, acidic condition prefers the 2e^−^ pathway to generate H_2_O_2_ and alkaline condition prefers the 4e^−^ pathway to generate H_2_O. As shown in Table 2, as pH increases from 3.6 to 7.2 and 12.6, the value of n increases from 2.86 to 3.04 and 3.50 for the CoN_4_-HMC system, and n increases from 2.58 to 2.78 and 3.36 for CoN_3_C_1_-HMC system. Be it an acidic or alkaline condition, changing the coordination from CoN_4_-HMC to CoN_3_C_1_-HMC inhibits the selectivity to H_2_O; in another word, it enhances the selectivity to H_2_O_2_.

### 4.4. Mechanism Analysis

In these three studies, the researchers employed a variety of advanced experimental characterization techniques (particularly in situ/operando spectroscopic methods) and computational studies to investigate the mechanism of the ORR catalyzed by asymmetric CoN_3_C_1_ sites. Although all three studies agree that breaking the symmetry by introducing a carbon atom enhances the catalytic performance, they differ in their emphasis regarding the mechanistic details observed experimentally and the interpretation of the performance improvement.

**Figure 8:**
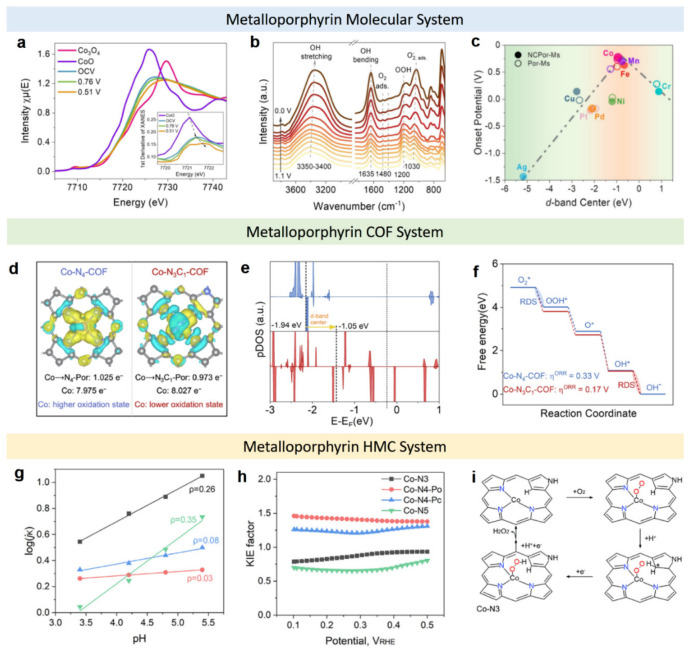
Mechanism studies of N-confused metalloporphyrin ORR catalysts in molecular (**a**–**c**), COF (**d**–**f**), and HMC systems (**g**–**i**). (**a**) Co K-edge XANES spectra of CoN_3_C_1_-TPP under different applied potentials. (**b**) In situ FTIR spectra of CoN_3_C_1_-TPP during the ORR process. (**c**) Relationship between onset potential and d-band center for M-N_3_C_1_-TPP and M-N_4_-TPP molecular systems. Experiment condition in O_2_-satuated 0.1 M KOH (pH ≈ 13). (**d**) Charge density difference and oxidation-state comparison between CoN_4_-COF and CoN_3_C_1_-COF. (**e**) Projected density of states of CoN_4_-COF and CoN_3_C_1_-COF. (**f**) Free energy diagrams of ORR elementary steps on CoN_4_-COF and CoN_3_C_1_-COF. (**g**) pH-dependent ORR kinetic behavior of CoN_3_C_1_-HMC and related Co coordination structures. (**h**) KIE factors of CoN_3_C_1_-HMC and related Co coordination structures at different potentials. (**i**) Proposed ORR mechanism on CoN_3_C_1_-HMC. Adapted from reference [49,50,52].

Huang et al. focused on the oxidation evolution of Co and intermediates during the ORR process of the molecular catalysts. The in situ XAS analysis indicates the oxidation of partial Co(II) within the CoN_3_C_1_-TPP sites to Co(III) as the catalytic center in the ORR process. The Co K-edge XANES spectra of CoN_3_C_1_-TPP showed a gradual shift in both the edge position and the shoulder to higher energy as the potential decreased from OCV to 0.51 V, which is close to that of Co_3_O_4_ (Figure 8a). And the in situ ATR-SEIRAS shows that vibrational signals associated with *O_2_ (~1480 cm^−1^ for O-O stretching) and *OOH (~1200 cm^−1^ for O-O stretching) can be observed on CoN_3_C_1_ sites, whereas *HOOH-related features (~1430 cm^−1^) are more readily detected on CoN_4_-Por (Figure 8b). The latter indicates the low selectivity for the 4e^−^ pathway on CoN_4_-Por.

The enhanced ORR reactivity and selectivity in CoN_3_C_1_-Por molecule were attributed to the following reasons. First, CoN_3_C_1_-TPP molecule has stronger electron donation capability than the CoN_4_-Por molecule, for the former has a higher HOMO level and a lower HOMO-LUMO gap, supported by both DFT calculations, synchrotron UPS measurements, and UV absorption. Secondly, the CoN_3_C_1_-TPP molecule has a higher redox capability, as the experimental CV curves indicate it has a lower initial oxidation potential and higher initial reduction potential than CoN_4_-Por. Third, the Co site in CoN_3_C_1_-TPP is more electron-rich than that in the CoN_4_-Por molecule, supported by electrostatic potential (ESP) and differential charge density calculations by the DFT method. Fourth, the d-band center of CoN_3_C_1_-TPP (−0.94 eV) is closer to the Fermi level (*E*_f_) than that of the CoN_4_-TPP molecule (−0.97 eV), so that CoN_3_C_1_-TPP can exhibit stronger absorption for the reaction intermediates. Note that the plots of theoretical *E*_onset_ of various metal centers against the d-band center display a volcano shape and the Co-systems are on the peak (Figure 8c). Analysis of each Co d-orbital supports that CoN_3_C_1_-TPP has stronger adsorption for O_2_ and *OOH, while its adsorption for *O is weaker. Fifth, the DFT-calculated free energy profiles (Figure 6b) showed that the formation of *OOH is the potential limiting step and the CoN_3_C_1_-TPP molecule has a lower limiting potential than the CoN_4_-Por molecule. Hence, there is a lower calculated *η*^ORR^ of 0.46 eV (CoN_3_C_1_-TPP) than 0.50 eV (CoN_4_-TPP). Sixth, the free energy for the formation of *OH, i.e., ∆*G*_O*→*OH_, is more negative for CoN_3_C_1_-TPP (−0.93 eV) than CoN_4_-TPP (−0.82 eV), so the asymmetric system exhibits higher selectivity in the 4e^−^ pathway.

In the COF work, using electron paramagnetic resonance (EPR) spectroscopy combined with the DMPO trapping agent, a typical DMPO-OH radical adduct was detected in the Co N_3_C_1_-COF system, whereas the DMPO-OOH adduct was detected in the metal-free COF. This confirms that the Co site is key to driving the 4e^−^ reduction to produce water. The XPS (the low-field shift in the Co 2p binding energy) and XANES (the red shift in the pre-edge and the position of the absorption edge) demonstrate that Co in CoN_3_C_1_-COF is in a lower oxidation state (i.e., higher electron density) than that in Co(II)N_4_-COF. This is consistent with the periodic DFT calculations in this work where Co transfers less electrons to the framework (Figure 8d), as well as the cluster DFT calculations [50]. Similarly to the work by Huang et al., the more electron-rich Co and the raised d-band center in CoN_3_C_1_-system lead to a larger overlap between the d-orbitals of Co and the empty p orbitals of the O atoms of both *O_2_ and *OOH moieties, thus stabilizing the *O_2_ and *OOH (Figure 8e). Shao et al. further argue that this leads to more balanced adsorption strengths, thus helping lower the overpotential, namely 0.17 V for CoN_3_C_1_-COF and 0.33 V for CoN_4_-Por-COF, as calculated without applied potential (Figure 8f).

The HMC work primarily focused on the 2e^−^ ORR pathway in acidic condition to produce H_2_O_2_ and proposed a new mechanism that differs from the previous two studies. The thiocyanate poisoning tests indicated that CoN_3_C_1_-HMC has a stronger binding to O_2_ than does CoN_4_-HMC. The ORR reaction order to H^+^ in acidic condition (pH from 3.6 to 5.4) was found to be zero for CoN_4_-HMC, in contrast to ρ = 0.236 for CoN_3_C_1_-HMC, indicating different pathways for the participation of H^+^ (Figure 8g). The ORR kinetic test conducted in H_2_O and D_2_O electrolytes revealed an isotope effect for CoN_4_-HMC (KIE_H/D_ = 1.2–1.4) and an inversed isotope effect for CoN_3_C_1_-HMC (KIE_H/D_ = 0.79–0.93), implying proton incorporation in the rate determining step of the latter (Figure 8h). Hence, this work proposed a novel mechanism. In the asymmetric configuration, the first coordination shell can be temporarily protonated (forms C-H or N-H) before the proton is transferred to *O_2_ intermediates, and these protonated sites act as a “proton-relay” station to accelerate the reaction kinetics (Figure 8i).

Huang et al. used the following observations to support this novel mechanism. First, the operando Co K-edge XANES spectra exhibit blue-shifted edge energies after biasing to 0.4 V_RHE_, suggesting that the Co valence increases after oxygenous intermediates’ adsorption. We note that the work by Huang et al. observed the same shift in the CoN_3_C_1_ molecular catalyst and they also ascribed it to the valence increase in Co(II) to Co(III). Second, operando WT-EXAFS showed that, at 0.4 VRHE, the first-shell Co-N path underwent a further contraction, with the R value decreasing from 1.412 to 1.376 Å, whereas the Co-C path became weaker, indicating a local structural response of the asymmetric CoN_3_ site under working conditions. Third, the FTIR spectra of CoN_3_C_1_-HMCs in the HClO_4_/H_2_O electrolyte shows that a new peak at about 2985 cm^−1^ was observed under 0.2 V_RHE_, and it was absent under dry state. This new peak was assigned to a sp^3^ C-H stretching mode and was attributed to C-atom protonation. This mode assignment was further confirmed by conducting tests in deuterated electrolytes (DClO_4_/D_2_O), where the presumed sp^3^ C-H stretching shifted to 2104 cm^−1^, reflecting the effect of isotope substitution. We have the opinion that the formation of the C-H bond on the confused C-atom during the ORR process is critically important. Those intermediates have not been considered in all the theoretical computations on the reaction mechanisms of the CoN_3_C_1_ system, including the work by Liu et al. This mechanism merits detailed computational investigation.

Taken together, these three studies support a common mechanistic picture in which first-coordination-sphere asymmetry enhances ORR activity by redistributing the electronic structure around the Co center, which means the N→C substitution increases the electron density at the Co site, thereby improving the adsorption and conversion of key oxygenated intermediates, especially *O_2_ and *OOH. In the molecular and COF systems, this electronic effect is further translated into more favorable 4e^−^ reduction, as reflected by easier *OOH formation and subsequent conversion, lower overpotentials, and higher H_2_O selectivity. In contrast, in the CNT-supported HMC system, the asymmetric CoN_3_C_1_ site still exhibits higher ORR activity and stronger O_2_ binding, but its mechanistic role is more strongly coupled to proton participation and operando structural response, leading to a proton-relay-assisted 2e^−^ pathway toward H_2_O_2_ under acidic conditions. Therefore, the three studies are consistent in identifying first-coordination-sphere inversion as the electronic origin of activity enhancement, while the final 2e^−^/4e^−^ selectivity remains dependent on the catalyst construction mode and local reaction environment.

We need to acknowledge that there are limitations in the current mechanistic understanding. In terms of experiment, the identification of key intermediates, such as *OOH, *O, and *OH in these metalloporphyrin systems, remains largely indirect. These species are inferred from electrochemical behavior, vibrational spectra, or X-ray absorption responses. It would be ideal to isolate stable intermediates and resolve their structures crystallographically [110]. The in situ and operando techniques still face important limitations like overlapping signals, insufficient temporal resolution, and limited structural specificity [111,112]. For example, the assignment of the CoN_3_C_1_ structure and the protonation site under the acidic condition remain to be resolved. In terms of computational studies, above results are mainly based on the thermodynamics data as computed from the periodic DFT method and the CHE model. Of note, under this framework, several important effects are neglected. For example, it lacks explicit treatment of solvent and interfacial effects, where the dynamics of solvent molecules, protons, and alkali ions at the microenvironment of the interface can be important. In addition, the reaction process may be kinetically controlled, and the protonation and electron-transfer steps may be sequential. The PCET treatment without computing the transition states may not be sufficient. For studies that capture the kinetics and the solvent, dynamical and interfacial effects are anticipated.

### 4.5. Comparison Between Modification Methods

Herein, we compare the effect and mechanism of the N-confused strategy to other four strategies discussed in Section 3. Table 1 and Table 2 list the exact values.

In terms of the effect on catalytic activity, N-confused strategy makes the *E*_1/2_ shift to positive by 0.06–0.12 V. This is comparable to the effect of meso-position modification, where the shift in *E*_1/2_ is 0.01–0.11 V, and where simple electron-donating substitution usually gives only 0.01–0.02 V, while the optimization of spatial arrangement can reach 0.11 V. Axial coordination can increase the *E*_1/2_ by about 0.13 V. The dinuclear strategy gives the largest improvement—a shift in *E*_1/2_ by 0.31 V. We note that the effect of β-position modification is reflected in selectivity.

In terms of selectivity, the N-confused strategy improves the 4e^−^ ORR preference in both the molecular and COF systems. The H_2_O_2_ yield decreases to <10% for CoN_3_C_1_-TPP, and in the COF system, n increases from 3.2 to about 3.8 while the H_2_O_2_ yield decreases from 22% to <10%. This effect is comparable to several other modification strategies. For *meso*-position modification, CoTPPy increases n from 2.6 to 3.51 through a proton-relay effect, and CoTPPNMe_3_^+^ gives n values of 3.2–3.8. For β-position modification, β-fluorinated cobalt porphyrins show high H_2_O selectivity, with n values of 3.84–3.94. Axial coordination increases n from 2.78 to 3.29 in the CoTPFPP/CoTPFPP-Im pair, while the dinuclear strategy gives a larger improvement, increasing n from 2.90 to 3.90 in the CoTPP/CoTPP-CoTPFPP comparison. From a mechanistic perspective, the N-confused strategy increases the electron density of the Co center, thereby the followed by O_2_ activation and the conversion of key intermediates. This is similar to the effect of *meso*-position modification (by electron-donating substitution) [99] and axial coordination (by electro-pushing effect) [36,103]. On the other hand, the possible involvement of local proton relay at the asymmetric site proposed by Liu et al. under acidic conditions is conceptually similar to the proton-transfer-promoting microenvironment introduced by hangman porphyrins and cationic meso-substituents [52]. As seen, the asymmetric first coordination by the N-confused strategy has multiple functions in shaping the reaction mechanism towards ORR.

From a material design perspective, the creation of asymmetric first coordination by C, N conversion may be combined with other strategies. For example, introducing hangman, pyridyl, or cationic groups at meso-position, or introducing axial electron-pushing ligands to the N-confused system may further increase the Co electron density as well as optimize local proton transfer. Designing the N-confused system in the dinuclear porphyrin system adds another dimension of asymmetric; this may facilitate the O_2_ activation during the O-O bond cleavage step. Although these combinations may yield new cobalt porphyrin catalysts with improved performance, they also increase synthetic complexity, as catalyst stability is critically important. Anchoring the synthesized molecular catalysts on carbon nanotube via van der Walls interaction could be a good strategy to maintain its stability.

## 5. Summary and Outlook

We reviewed recent progress of N-confused cobalt porphyrin catalysts for electro-catalyzing the oxygen reduction reaction, focusing on how breaking the N_4_ symmetry affects catalytic reactivity, the selectivity of ORR, and the underlying mechanisms. Experimentally, CoN_3_C_1_ porphyrin-based catalysts were synthesized in three architectures, i.e., molecular catalysts, covalent organic frameworks (COFs), and CNT-supported heterogeneous molecular catalysts (HMCs). All reported that the CoN_3_C_1_ systems are more active than the corresponding CoN_4_ systems. Based on the reported experimental measured E_1/2_ and *E*_onset_ potentials, the CoN_3_C_1_-Por molecular catalysts display the best ORR performance.

Mechanistically, the common origin of this enhancement lies in the redistribution of the electronic structure around the Co site: the Co center generally becomes more electron-rich, the d-band center shifts closer to the Fermi level, and the interaction with O_2_ and key oxygenated intermediates is consequently altered. At the same time, these studies show that selectivity depends on the form of the catalysts. Under basic condition, all systems prefer the 4e^−^ pathway over 2e^−^ pathway. Changing the coordination from CoN_4_ to CoN_3_C_1_ improves 4e^−^ ORR selectivity in both molecular and COF systems, but decreases 4e^−^ selectivity in the CNT-supported HMC system. The underlying mechanisms have not been elucidated yet; hence, it merits further studies. In addition, we note that in all the computational studies, proton transfer and electron transfer were treated simultaneously, and the kinetics were not considered, i.e., no transition states were computed. A recent paper by Liao group provides a good reference for the model and method [94]. We anticipate these aspects to be addressed in future works. This could be an important step to bridge the gap between heterogeneous molecular mechanisms and homogeneous materials.

The effects of N-confused strategies on ORR are comparable to other more commonly used modification strategies, including meso- and β-position substitution, axial coordination, and dinuclear design. They affected one or several key issues: how to tune the electronic structure of the Co center, how to optimize the local proton-transfer environment, and how to modify the stability of key oxygen-containing intermediates and the process of O-O bond cleavage.

In brief, breaking the N_4_ symmetry by inverting the C, N atoms proved to be an effective strategy to improve ORR electrocatalytic activity and selectivity. We propose the following aspects for future studies: First, combine the N-confused strategy with other modification strategies to expand the types of catalysts so that to explore possible synergistic effects. Second, investigate the effect of architecture of catalysts for a better design of heterogeneous materials that can be used in fuel cell batteries. Third, for computational studies, it is urgent to explore beyond the PCET assumptions and gain a complete understanding of the mechanisms. The development of N-confused metalloporphyrin catalysts could be significantly improved with continued advances in multi-strategy catalyst design and synthesis, as well as in-depth mechanistic studies.

## Figures and Tables

**Table 1:** Cobalt porphyrin-based catalyst systems and their electro-catalyzing performance towards ORR.

No.	Catalysts	*^a^* Modify	*^b^* Support	Solvent	*^c^* E_1/2_ (V vs. RHE)	*^d^* n	Ref.
**1**	CoTPP	-	CNT	0.1M KOH	0.81 V	/	[98]
**2**	CoTPFPP	*meso*			0.76 V	/	
**1**	CoTPP	*-*	GCE	1M HClO_4_	0.55 V	2.0	[99]
**3**	CoTMePP	*meso*			0.56 V	2.5	
**4**	CoTMeOPP	*meso*			0.57 V	3.3	
**1**	CoTPP	*-*	EPG	1M H_2_SO_4_	0.5–0.6 V_NHE_ (E_onset_)	2.6	[100]
**5**	CoTPPy	*meso*			0.6–0.7 V_NHE_ (E_onset_)	3.51	
**6**	CoTPPNH_2_	*meso*	EPG	H_2_SO_4_/NaOH (pH = 0/4/7)	/	3.1/2.7/3.0	[45]
**7**	CoTPPNMe_2_				/	3.4/3.0/3.1	
**8**	CoTPPNMe_3_^+^				/	3.8/3.5/3.2	
**9**	amido-CoTPP(αβαβ)	*meso*	CNT	0.1M KOH	0.73 V	2.10	[47]
**10**	amido-CoTPP(αααα)				0.72–0.73 V	3.75	
**11**	amido-CoTPP(ααββ)				0.72–0.73 V	2.89–3.10	
**12**	amido-CoTPP(αααβ)				0.72–0.73 V	2.89–3.10	
**13**	aBz-TCoP	*meso*	CB	0.5M H_2_SO_4_/ 0.1M KOH	0.41 V/ 0.70 V	3.2 3.0	[101]
**14**	Bz-2TCoP				0.52 V/ 0.77 V	3.6 3.5	
**15**	CoTP(OH)_2_	*β*	-	0.13M AcOH	−1.36 V_Fc/Fc+_	-	[102]
**16**	CoTPF_8_(OH)_2_				−0.97 V_Fc/Fc+_	3.84	
**17**	CoTPF_8_(OH)_4_				−1.01 V_Fc/Fc+_	3.94	
**18**	(T*p*YPP)Co	*β*	EPG	1M HClO_4_	/	2.4	[33]
**19**	Butano-(T*p*YPP)Co				/	2.0	
**20**	Benzo-(T*p*YPP) Co				/	2.6–3.1	
**21**	MPy-TMPPCo	*axial*	Au	0.5M H_2_SO_4_	0.53 V (E_onset_)	~2	[36]
**22**	APT-TMPPCo				0.48 V (E_onset_)	~2	
**23**	MBN-TMPPCo				0.45 V (E_onset_)	~2	
**2**	CoTPFPP	*axial*	CNT	1.0 M KOH	0.70 V	2.78	[103]
**24**	CoTPFPP-Im				0.83 V	3.29	
**1**	CoTPP	-	CNT	0.5 M H_2_SO_4_	0.41 V	2.90	[46]
**2**	CoTPFPP	-			0.46 V	2.90	
**25**	CoTPP/CoTPFPP	dinuclear			0.72 V	3.90	
**26**	(CoTPFPP)_2_	dinuclear			0.55 V	3.80	
**27**	(CoTPyP)_2_	dinuclear	GCE	0.5 M H_2_SO_4_	0.16 V _Ag/AgCl_	~3.00	[104]
**28**	(Co-Tolyl)_2_				0.38 V _Ag/AgCl_	~3.97	
**29**	(Co-Phenyl)_2_				0.35 V _Ag/AgCl_	~3.93	
**30**	(Co-Chloro)_2_				0.37 V _Ag/AgCl_	~3.79	
**31**	(Co-CF_3_)_2_				0.37 V _Ag/AgCl_	~3.85	
	CoN_4_-TPP	-	GCE	0.1 M KOH	0.77 V	3.85–3.95	[49]
	CoN_3_C_1_-TPP	first coord			0.83 V	3.75–3.90	
	CoN_4_-COF	-	COF	0.1 M KOH	0.675 V	3.8	[50]
	CoN_3_C_1_-COF	first coord			0.794 V	3.2	
	CoN_4_-HMC	-	CNT	0.1 M ABS 0.1 M PBS 0.1 M KOH	0.682 V 0.68 V 0.85 V	2.58 2.78 3.36	[53]
	CoN_3_C_1_-HMC	first coord	CNT		0.747 V 0.73 V 0.93 V	2.86 3.04 3.50	

*^a^* Modify refers to the modification strategies. *^b^* Support refers to the support material during electrochemistry tests. CNT means carbon nanotube, EPG means edge-plane graphite, GCE means glassy carbon electrode, and CB means carbon black. *^c^* Unless otherwise specified, all reported potentials are half-wave potentials (E_1/2_) referenced to RHE. *^d^* n refers to the number of electrons transferred during ORR.

**Table 2:** Comparison of experimental and theoretical ORR performance of CoN_4-_ and CoN_3_C-active sites in N-confused cobalt porphyrin-based catalysts under different electrolyte environments and support platforms.

Catalysts	Electrolytes	pH	Electrochem. Method	*^a^* E_1/2_ (V vs. RHE)	*^b^* E_onset_ (V vs. RHE)	*^c^* n	*^d^* H_2_O_2_%	*^e^ η*_ORR_ (V, DFT)	*^b^* E_onset_ (V, DFT)	*^f^* d-Band Center	*^g^* PDS	Ref.
CoN_4_-TPP	0.1 M KOH	13	*^h^* RDE-LSV/RRDE	0.77 V	0.84 V	3.75–3.90	6–12%	0.50	0.73	−0.97 eV	*OOH	[49]
CoN_3_C_1_-TPP	0.1 M KOH	13		0.83 V	0.95 V	3.85–3.95	<10%	0.46	0.77	−0.94 eV	*OOH	
CoN_4_-COF	0.1 M KOH	/	RDE-LSV/RRDE	0.675 V	0.82 V	3.2	22%	0.33	0.90	-	*OOH	[50]
CoN_3_C_1_-COF	0.1 M KOH	/		0.794 V	0.89 V	3.8	<10%	0.17	1.06	-	*OOH	
CoN_4_-HMC	0.1 M *^h^* ABS	3.6	RRDE-LSV	0.45 V	0.682 V	2.86	72.5%	0.485 (2e)	0.745	−0.78 eV	-	[52]
	0.1 M *^h^* PBS	7.2		0.45 V	0.68 V	3.04	48%	0.561 (4e-associative)	0.669			
	0.1 M KOH	12.6		0.60 V	0.85 V	3.50	25%	0.601 (dissociative)	0.629			
CoN_3_C_1_-HMC	0.1 M ABS	3.6	RRDE-LSV	0.62 V	0.747 V	2.58	83.0%	0.597 (all pathway)	0.633	−0.68 eV	-	
	0.1 M PBS	7.2		0.65 V	0.73 V	2.78	61%					
	0.1 M KOH	12.6		0.78 V	0.93 V	3.36	32%					
Pt/C	0.1 M KOH	13	RDE-LSV/RRDE	*^i^* 0.81 V	*^i^* 0.92 V	-	-	-	-	-	-	[49]

*^a^* E_1/2_ refers to the half-wave potential referenced to the reversible hydrogen electrode (RHE). *^b^* E_onset_ refers to the onset potential referenced to the reversible hydrogen electrode (RHE); unlabeled values are experimental, whereas values labeled “DFT” are theoretically calculated. *^c^* n refers to the number of electrons transferred during ORR. *^d^* H_2_O_2_% refers to the percentage yield or selectivity of hydrogen peroxide during ORR. *^e^* ηORR refers to the theoretical ORR overpotential calculated by density functional theory (DFT). *^f^* d-band center refers to the center position of the metal d-band relative to the Fermi level (Ef). *^g^* PDS refers to the potential-determining step. *^h^* ABS, acetate-buffered saline; PBS, phosphate-buffered saline; RRDE-LSV, linear sweep voltammetry using a rotating ring-disk electrode; RDE-LSV, linear sweep voltammetry using a rotating disk electrode. *^i^* estimated from Figure 2b in the original article by Huang et al. [49].

## Data Availability

No new data were created or analyzed in this study. Data sharing is not applicable to this article.

## References

[1] Debe M.K. (2012). Electrocatalyst Approaches and Challenges for Automotive Fuel Cells. Nature.

[2] Shao M., Chang Q., Dodelet J.-P., Chenitz R. (2016). Recent Advances in Electrocatalysts for Oxygen Reduction Reaction. Chem. Rev..

[3] Kulkarni A., Siahrostami S., Patel A., Nørskov J.K. (2018). Understanding Catalytic Activity Trends in the Oxygen Reduction Reaction. Chem. Rev..

[4] Poudel M.B., Anand R., Ojha G.P., Kim A.R., Kumar R.S., Bhandari T., Sakthivel V., Yun G.J., Yoo D.J. (2026). Nitrogen Coordinated Atomically Dispersed Manganese Catalyzes Oxygen Reduction in Practical Zinc-Air Batteries and Alkaline Fuel Cells. J. Power Sources.

[5] Nørskov J.K., Rossmeisl J., Logadottir A., Lindqvist L., Kitchin J.R., Bligaard T., Jónsson H. (2004). Origin of the Overpotential for Oxygen Reduction at a Fuel-Cell Cathode. J. Phys. Chem. B.

[6] Zhang J., Yuan Y., Gao L., Zeng G., Li M., Huang H. (2021). Stabilizing Pt-Based Electrocatalysts for Oxygen Reduction Reaction: Fundamental Understanding and Design Strategies. Adv. Mater..

[7] Niu W., Pakhira S., Cheng G., Zhao F., Yao N., Mendoza-Cortes J.L., Koel B.E. (2024). Reaction-Driven Restructuring of Defective PtSe2 into Ultrastable Catalyst for the Oxygen Reduction Reaction. Nat. Mater..

[8] Bashyam R., Zelenay P. (2006). A Class of Non-Precious Metal Composite Catalysts for Fuel Cells. Nature.

[9] Lefèvre M., Proietti E., Jaouen F., Dodelet J.-P. (2009). Iron-Based Catalysts with Improved Oxygen Reduction Activity in Polymer Electrolyte Fuel Cells. Science.

[10] Liu K., Fu J., Lin Y., Luo T., Ni G., Li H., Lin Z., Liu M. (2022). Insights into the Activity of Single-Atom Fe-N-C Catalysts for Oxygen Reduction Reaction. Nat. Commun..

[11] Ao X., Wang H., Zhang X., Wang C. (2025). Atomically Dispersed Metal–Nitrogen–Carbon Catalysts for Acidic Oxygen Reduction Reaction. ACS Appl. Mater. Interfaces.

[12] Xu H., Wang D., Yang P., Liu A., Li R., Li Y., Xiao L., Ren X., Zhang J., An M. (2020). Atomically Dispersed M–N–C Catalysts for the Oxygen Reduction Reaction. J. Mater. Chem. A.

[13] Collman J.P., Devaraj N.K., Decréau R.A., Yang Y., Yan Y.-L., Ebina W., Eberspacher T.A., Chidsey C.E.D. (2007). A Cytochrome c Oxidase Model Catalyzes Oxygen to Water Reduction Under Rate-Limiting Electron Flux. Science.

[14] Shyu T.C., Damasceno P.F., Dodd P.M., Lamoureux A., Xu L., Shlian M., Shtein M., Glotzer S.C., Kotov N.A. (2015). A Kirigami Approach to Engineering Elasticity in Nanocomposites through Patterned Defects. Nat. Mater..

[15] Wang A., Li J., Zhang T. (2018). Heterogeneous Single-Atom Catalysis. Nat. Rev. Chem..

[16] Collman J.P., Boulatov R., Sunderland C.J., Fu L. (2004). Functional Analogues of Cytochrome c Oxidase, Myoglobin, and Hemoglobin. Chem. Rev..

[17] Zhang W., Lai W., Cao R. (2017). Energy-Related Small Molecule Activation Reactions: Oxygen Reduction and Hydrogen and Oxygen Evolution Reactions Catalyzed by Porphyrin- and Corrole-Based Systems. Chem. Rev..

[18] Wikström M., Krab K., Sharma V. (2018). Oxygen Activation and Energy Conservation by Cytochrome c Oxidase. Chem. Rev..

[19] Nolfi-Donegan D., Braganza A., Shiva S. (2020). Mitochondrial Electron Transport Chain: Oxidative Phosphorylation, Oxidant Production, and Methods of Measurement. Redox Biol..

[20] Zhao J., Wu Y., Liu C., Zhang B., Gao Y. (2024). Enzyme-Inspired Molecular Electrocatalysts for the Oxygen Reduction Reaction. Electrochim. Acta.

[21] Ortiz De Montellano P.R. (2015). Cytochrome P450: Structure, Mechanism, and Biochemistry.

[22] Grimm B., Porra R.J., Rüdiger W., Scheer H. (2006). Chlorophylls and Bacteriochlorophylls.

[23] Warren M.J., Raux E., Schubert H.L., Escalante-Semerena J.C. (2002). The Biosynthesis of Adenosylcobalamin (Vitamin B12). Nat. Prod. Rep..

[24] Li X., Lei H., Xie L., Wang N., Zhang W., Cao R. (2022). Metalloporphyrins as Catalytic Models for Studying Hydrogen and Oxygen Evolution and Oxygen Reduction Reactions. Acc. Chem. Res..

[25] Liang Z., Wang H.-Y., Zheng H., Zhang W., Cao R. (2021). Porphyrin-Based Frameworks for Oxygen Electrocatalysis and Catalytic Reduction of Carbon Dioxide. Chem. Soc. Rev..

[26] Li Z., Wei Q., Ren Z., Xie J. (2025). Recent Progress in Heteroatom-Containing Metalloporphyrin-Based Catalysts for CO_2_ Reduction. Molecules.

[27] Dong Y., Lv X., Sun Y., Zhao Q., Lei H., Wu F., Zhang T., Xue Z., Cao R., Qiu F. (2024). Electrocatalytic Oxygen Reduction Reaction of Peripheral Functionalized Cobalt Porphyrins(2.1.2.1). Inorg. Chem..

[28] Xu Q., Zhao L., Ma Y., Yuan R., Liu M., Xue Z., Li H., Zhang J., Qiu X. (2021). Substituents and the Induced Partial Charge Effects on Cobalt Porphyrins Catalytic Oxygen Reduction Reactions in Acidic Medium. J. Colloid Interface Sci..

[29] Yuan R., George S.L., Chen J., Wu Q., Qiu X., Zhao L. (2023). Meso-Substituted Metalloporphyrin-Based Composites for Electrocatalytic Oxygen Reduction Reactions. ChemNanoMat.

[30] Brezny A.C., Johnson S.I., Raugei S., Mayer J.M. (2020). Selectivity-Determining Steps in O_2_ Reduction Catalyzed by Iron(Tetramesitylporphyrin). J. Am. Chem. Soc..

[31] Rigsby M.L., Wasylenko D.J., Pegis M.L., Mayer J.M. (2015). Medium Effects Are as Important as Catalyst Design for Selectivity in Electrocatalytic Oxygen Reduction by Iron–Porphyrin Complexes. J. Am. Chem. Soc..

[32] Lei H., Li X., Meng J., Zheng H., Zhang W., Cao R. (2019). Structure Effects of Metal Corroles on Energy-Related Small Molecule Activation Reactions. ACS Catal..

[33] Ye L., Fang Y., Ou Z., Xue S., Kadish K.M. (2017). Cobalt Tetrabutano- and Tetrabenzotetraarylporphyrin Complexes: Effect of Substituents on the Electrochemical Properties and Catalytic Activity of Oxygen Reduction Reactions. Inorg. Chem..

[34] D’Souza F., Hsieh Y.-Y., Deviprasad G.R. (1997). Electrocatalytic Reduction of Molecular Oxygen Using Non-Planar Cobalt Tetrakis-(4-Sulfonatophenyl)-β-Octabromoporphyrin. J. Electroanal. Chem..

[35] Samanta S., Das P.K., Chatterjee S., Sengupta K., Mondal B., Dey A. (2013). O_2_ Reduction Reaction by Biologically Relevant Anionic Ligand Bound Iron Porphyrin Complexes. Inorg. Chem..

[36] Zhou Y., Xing Y.-F., Wen J., Ma H.-B., Wang F.-B., Xia X.-H. (2019). Axial Ligands Tailoring the ORR Activity of Cobalt Porphyrin. Sci. Bull..

[37] Xie L., Zhang X.-P., Zhao B., Li P., Qi J., Guo X., Wang B., Lei H., Zhang W., Apfel U.-P. (2021). Enzyme-Inspired Iron Porphyrins for Improved Electrocatalytic Oxygen Reduction and Evolution Reactions. Angew. Chem. Int. Ed..

[38] Cichocka M.O., Liang Z., Feng D., Back S., Siahrostami S., Wang X., Samperisi L., Sun Y., Xu H., Hedin N. (2020). A Porphyrinic Zirconium Metal–Organic Framework for Oxygen Reduction Reaction: Tailoring the Spacing between Active-Sites through Chain-Based Inorganic Building Units. J. Am. Chem. Soc..

[39] Cao Y., Mou Y., Zhang J., Zhang R., Liang Z. (2024). Porphyrin-Based Frameworks and Derivatives for the Oxygen Reduction Reaction. Mater. Today Catal..

[40] Lei H., Han J., Zhao Q., Liu J., Yan L., Zhang W., Cao R. (2025). Metalloporphyrin- and Metallocorrole-Based Catalysts for the Oxygen Reduction Reaction: From Molecules to Materials. Chem. Soc. Rev..

[41] Cheng N., Kemna C., Goubert-Renaudin S., Wieckowski A. (2012). Reduction Reaction by Porphyrin-Based Catalysts for Fuel Cells. Electrocatalysis.

[42] Kingsbury C.J., Senge M.O. (2021). The Shape of Porphyrins. Coord. Chem. Rev..

[43] Roucan M., Kielmann M., Connon S.J., Bernhard S.S.R., Senge M.O. (2017). Conformational Control of Nonplanar Free Base Porphyrins: Towards Bifunctional Catalysts of Tunable Basicity. Chem. Commun..

[44] Ishizuka T., Grover N., Kingsbury C.J., Kotani H., Senge M.O., Kojima T. (2022). Nonplanar Porphyrins: Synthesis, Properties, and Unique Functionalities. Chem. Soc. Rev..

[45] Zhang R., Warren J.J. (2020). Controlling the Oxygen Reduction Selectivity of Asymmetric Cobalt Porphyrins by Using Local Electrostatic Interactions. J. Am. Chem. Soc..

[46] Liu Y., Zhou G., Zhang Z., Lei H., Yao Z., Li J., Lin J., Cao R. (2019). Significantly Improved Electrocatalytic Oxygen Reduction by an Asymmetrical Pacman Dinuclear Cobalt(II) Porphyrin–Porphyrin Dyad. Chem. Sci..

[47] Lv B., Li X., Guo K., Ma J., Wang Y., Lei H., Wang F., Jin X., Zhang Q., Zhang W. (2021). Controlling Oxygen Reduction Selectivity through Steric Effects: Electrocatalytic Two-Electron and Four-Electron Oxygen Reduction with Cobalt Porphyrin Atropisomers. Angew. Chem. Int. Ed..

[48] Ren Z., Gong K., Zhao B., Chen S.-L., Xie J. (2024). Boosting the Catalytic Performance of Metalloporphyrin-Based Covalent Organic Frameworks via Coordination Engineering for CO2 and O2 Reduction. Mater. Chem. Front..

[49] Huang S., Tranca D., Rodríguez-Hernández F., Zhang J., Lu C., Zhu J., Liang H.-W., Zhuang X. (2024). Well-Defined N_3_ C_1_ -Anchored Single-Metal-Sites for Oxygen Reduction Reaction. Angew. Chem. Int. Ed..

[50] Shao P., Ren Z., Zhao B., Wang X., Li J., Xie J., Wang B., Feng X. (2025). Theory-Guided Design of N-Confused Porphyrinic Covalent Organic Frameworks for Oxygen Reduction Reaction. J. Am. Chem. Soc..

[51] Liu T., Zhang Q., Guo H., Liang Z., Cao R. (2022). Electrocatalytic Oxygen Reduction Reaction with Metalloporphyrins. Sci. Sin.-Chim..

[52] Liu C., Zhang D., Chen J., She F., Liu F., Yu Z., Zheng Z., Levine M.S., Sessler J.L., Chen Y. (2026). Coordination-Dependent Oxygen Reduction Reaction Activity of Single Atom Co–N *x*–C Electrocatalysts. J. Am. Chem. Soc..

[53] Yan W., Cao S., Xiao Z., Dai F., Xing T., Li Z., Chen Y., Lu X., Li X. (2021). Novel Heteroatom Sulfur Porphyrin Organic Polymer as a Metal-Free Electrocatalyst for Acidic Oxygen Reduction Reaction. Electrochim. Acta.

[54] Yan W., Chen W., Chen Y. (2024). Recent Design Strategies for M-N-C Single-Atom Catalysts in Oxygen Reduction: An Entropy Increase Perspective. Adv. Funct. Mater..

[55] Qiu H., Wen S., Fu Q., Zhao X. (2025). Oxygen Reduction Reactions of Catalysts with Asymmetric Atomic Structures: Mechanisms, Applications, and Challenges. Catalysts.

[56] Cao P., Mu X., Chen F., Wang S., Liao Y., Liu H., Du Y., Li Y., Peng Y., Gao M. (2025). Breaking Symmetry for Better Catalysis: Insights into Single-Atom Catalyst Design. Chem. Soc. Rev..

[57] Toganoh M., Furuta H. (2022). Creation from Confusion and Fusion in the Porphyrin World─the Last Three Decades of N-Confused Porphyrinoid Chemistry. Chem. Rev..

[58] Harvey J.D., Ziegler C.J. (2003). Developments in the Metal Chemistry of N-Confused Porphyrin. Coord. Chem. Rev..

[59] Harvey J.D., Ziegler C.J. (2006). The Metal Complexes of N-Confused Porphyrin as Heme Model Compounds. J. Inorg. Biochem..

[60] Chatterjee T., Shetti V.S., Sharma R., Ravikanth M. (2017). Heteroatom-Containing Porphyrin Analogues. Chem. Rev..

[61] Thuita D.W., Brückner C. (2022). Metal Complexes of Porphyrinoids Containing Nonpyrrolic Heterocycles. Chem. Rev..

[62] Peng C.C., Yang F.-A., Chen J.-H., Wang S.-S., Tung J.-Y. (2008). Mercury(II) Complex of Inverted *N*-Methylated Porphyrin: HgPh(2-NCH_3_NCTPP). Polyhedron.

[63] Chmielewski P.J., Latos-Grażyński L. (1995). N-Methyltetraphenylporphyrin with an Inverted N-Methylpyrrole Ring: The First Isomer of N-Methyltetraphenylporphyrin. J. Chem. Soc. Perkin Trans. 2.

[64] Chmielewski P.J., Latos-Grażyński L., Schmidt I. (2000). Copper(II) Complexes of Inverted Porphyrin and Its Methylated Derivatives. Inorg. Chem..

[65] Maeda H., Osuka A., Ishikawa Y., Aritome I., Hisaeda Y., Furuta H. (2003). N-Confused Porphyrin-Bearing Meso-Perfluorophenyl Groups:  A Potential Agent That Forms Stable Square-Planar Complexes with Cu(II) and Ag(III). Org. Lett..

[66] Chen W.C., Hung C.H. (2001). Synthesis and Characterization of Iron N-Confused Porphyrins: Structural Evidences of Agostic Interaction. Inorg. Chem..

[67] Bohle D.S., Chen W.-C., Hung C.-H. (2002). Metal Oxidation Promoted C-H Activation in Manganese Complexes of N-Confused Porphyrin. Inorg. Chem..

[68] Harvey J.D., Shaw J.L., Herrick R.S., Ziegler C.J. (2005). The Synthesis of Isostructural Mo2+ Porphyrin and N-Confused Porphyrin Complexes. Chem. Commun..

[69] Srinivasan A., Toganoh M., Niino T., Osuka A., Furuta H. (2008). Synthesis of N-Confused Tetraphenylporphyrin Rhodium Complexes Having Versatile Metal Oxidation States. Inorg. Chem..

[70] Toganoh M., Ikeda S., Furuta H. (2007). Synthesis, Reactivity, and Properties of N-Fused Porphyrin Rhenium(I) Tricarbonyl Complexes. Inorg. Chem..

[71] Furuta H., Kubo N., Maeda H., Ishizuka T., Osuka A., Nanami H., Ogawa T. (2000). N-Confused Double-Decker Porphyrins. Inorg. Chem..

[72] Toganoh M., Niino T., Furuta H. (2008). Luminescent Au(III) Organometallic Complex of N-Confused Tetraphenylporphyrin. Chem. Commun..

[73] Furuta H., Maeda H., Osuka A. (2000). Doubly N-Confused Porphyrin:  A New Complexing Agent Capable of Stabilizing Higher Oxidation States. J. Am. Chem. Soc..

[74] Maeda H., Osuka A., Furuta H. (2003). Trans Doubly N-Confused Porphyrins:  Cu(III) Complexation and Formation of Rodlike Hydrogen-Bonding Networks. J. Am. Chem. Soc..

[75] Latos-Grazynski L., Lisowski J., Olmstead M.M., Balch A.L. (1989). Five-Coordinate Complexes of 21-Thiaporphyrin. Preparations, Spectra, and Structures of Iron(II), Nickel(II), and Copper(II) Complexes. Inorg. Chem..

[76] Pawlicki M., Latos-Grażyński L. (2002). Iron Complexes of 5,10,15,20-Tetraphenyl-21-Oxaporphyrin. Inorg. Chem..

[77] Chmielewski P.J., Latos-Grażyński L., Olmstead M.M., Balch A.L. (1997). Nickel Complexes of 21-Oxaporphyrin and 21, 23-Dioxaporphyrin. Chem.–Eur. J..

[78] Ghosh A., Ravikanth M. (2012). Rhenium(I) Tricarbonyl Complexes of 5,10,15,20-Tetraphenyl-21-Thia and 21-Oxaporphyrins. Inorg. Chem..

[79] Stute S., Götzke L., Meyer D., Merroun M.L., Rapta P., Kataeva O., Seichter W., Gloe K., Dunsch L., Gloe K. (2013). Molecular Structure, UV/Vis Spectra, and Cyclic Voltammograms of Mn(II), Co(II), and Zn(II) 5,10,15,20-Tetraphenyl-21-Oxaporphyrins. Inorg. Chem..

[80] Latos-Grazynski L., Lisowski J., Olmstead M.M., Balch A.L. (1989). Preparation and Structural Characterization of a Six-Coordinate 21-Thiaporphyrin Complex: RhIII(STPP)Cl2 (STPP = Tetraphenyl-21-Thiaporphyrin Anion). Inorg. Chem..

[81] Latos-Grazynski L., Lisowski J., Chmielewski P., Grzeszczuk M., Olmstead M.M., Balch A.L. (1994). Palladium Complexes of 21-Thiaporphyrin: Syntheses and Characterization. Inorg. Chem..

[82] Gebauer A., Schmidt J.A.R., Arnold J. (2000). Synthesis, Characterization, and Properties of a Lithium 21-Thiaporphyrin Complex. Inorg. Chem..

[83] Chuang C.-H., Ou C.-K., Liu S.-T., Kumar A., Ching W.-M., Chiang P.-C., Dela Rosa M.A.C., Hung C.-H. (2011). Ruthenium Complexes of Thiaporphyrin and Dithiaporphyrin. Inorg. Chem..

[84] Hung C.-H., Ou C.-K., Lee G.-H., Peng S.-M. (2001). Structure and Characterization of the First Metal Complex of Dithiaporphyrin:  Ru(S2TTP)Cl2. Inorg. Chem..

[85] Kaur T., Ghosh A., Rajakannu P., Ravikanth M. (2014). Synthesis and Crystal Structure of the Rhenium(I) Tricarbonyl Complex of 5,10,15,20-Tetra-p-Tolyl-21,23-Dithiaporphyrin. Inorg. Chem..

[86] Hung C.-H., Peng C.-H., Shen Y.-L., Wang S.-L., Chuang C.-H., Lee H.M. (2008). Preparation and Oxygenation of Cobalt N-Confused Porphyrin Nitrosyl Complexes. Eur. J. Inorg. Chem..

[87] Harvey J.D., Ziegler C.J. (2004). Dianionic and Trianionic Macrocycles in Cobalt N-Confused Porphyrin Complexes. Chem. Commun..

[88] Tian Z., Wang Y., Li Y., Yao G., Zhang Q., Chen L. (2022). Theoretical Study of the Effect of Coordination Environment on the Activity of Metal Macrocyclic Complexes as Electrocatalysts for Oxygen Reduction. iScience.

[89] Pegis M.L., Wise C.F., Martin D.J., Mayer J.M. (2018). Oxygen Reduction by Homogeneous Molecular Catalysts and Electrocatalysts. Chem. Rev..

[90] Pegis M.L., McKeown B.A., Kumar N., Lang K., Wasylenko D.J., Zhang X.P., Raugei S., Mayer J.M. (2016). Homogenous Electrocatalytic Oxygen Reduction Rates Correlate with Reaction Overpotential in Acidic Organic Solutions. ACS Cent. Sci..

[91] Fukuzumi S., Mochizuki S., Tanaka T. (1989). Efficient Reduction of Dioxygen with Ferrocene Derivatives, Catalyzed by Metalloporphyrins in the Presence of Perchloric Acid. Inorg. Chem..

[92] Siewert I. (2015). Proton-Coupled Electron Transfer Reactions Catalysed by 3 d Metal Complexes. Chem.–Eur. J..

[93] Dung T.P., Chihaia V., Son D.N. (2023). Effects of Functional Groups in Iron Porphyrin on the Mechanism and Activity of Oxygen Reduction Reaction. RSC Adv..

[94] Chen J.-Y., Cao Y.-C., Liao R.-Z. (2025). Computational Mechanistic Study of Oxygen Reduction by an Asymmetric Pacman Dicobalt Porphyrin Catalyst. ACS Catal..

[95] Limberg C. (2009). What Does It Really Take to Stabilize Complexes of Late Transition Metals with Terminal Oxo Ligands?. Angew. Chem. Int. Ed..

[96] Zhang X.-P., Chandra A., Lee Y.-M., Cao R., Ray K., Nam W. (2021). Transition Metal-Mediated O–O Bond Formation and Activation in Chemistry and Biology. Chem. Soc. Rev..

[97] Li Y., Wang N., Lei H., Li X., Zheng H., Wang H., Zhang W., Cao R. (2021). Bioinspired N4-Metallomacrocycles for Electrocatalytic Oxygen Reduction Reaction. Coord. Chem. Rev..

[98] Qin H., Wang Y., Wang B., Duan X., Lei H., Zhang X., Zheng H., Zhang W., Cao R. (2021). Cobalt Porphyrins Supported on Carbon Nanotubes as Model Catalysts of Metal-N4/C Sites for Oxygen Electrocatalysis. J. Energy Chem..

[99] Ardakani M.M., Rahimi P., Dehghani H., Karami P.E., Zare H.R., Karami S. (2007). Electrocatalytic Reduction of Dioxygen on the Surface of Glassy Carbon Electrodes Modified with Cobalt Porphyrin Complexes. Electroanalysis.

[100] Sinha S., Ghosh M., Warren J.J. (2019). Changing the Selectivity of O_2_ Reduction Catalysis with One Ligand Heteroatom. ACS Catal..

[101] Wei Y., Liang Y., Wu Q., Xue Z., Feng L., Zhang J., Zhao L. (2023). Effects of Tuning the Structural Symmetry of Cobalt Porphyrin on Electrocatalytic Oxygen Reduction Reactions. Dalton Trans..

[102] Chaturvedi A., Dash S., Sinha S., Panetier J.A., Jiang J. (2024). Effect of β-Fluorinated Porphyrin in Changing Selectivity for Electrochemical O2 Reduction. Mater. Today Catal..

[103] Li X., Li P., Yang J., Xie L., Wang N., Lei H., Zhang C., Zhang W., Lee Y.-M., Zhang W. (2023). A Cobalt(II) Porphyrin with a Tethered Imidazole for Efficient Oxygen Reduction and Evolution Electrocatalysis. J. Energy Chem..

[104] Crawley M.R., Zhang D., Oldacre A.N., Beavers C.M., Friedman A.E., Cook T.R. (2021). Tuning the Reactivity of Cofacial Porphyrin Prisms for Oxygen Reduction Using Modular Building Blocks. J. Am. Chem. Soc..

[105] McGuire R., Dogutan D.K., Teets T.S., Suntivich J., Shao-Horn Y., Nocera D.G. (2010). Oxygen Reduction Reactivity of Cobalt(II) Hangman Porphyrins. Chem. Sci..

[106] Chang C.J., Loh Z.-H., Shi C., Anson F.C., Nocera D.G. (2004). Targeted Proton Delivery in the Catalyzed Reduction of Oxygen to Water by Bimetallic Pacman Porphyrins. J. Am. Chem. Soc..

[107] Su J., Musgrave C.B., Song Y., Huang L., Liu Y., Li G., Xin Y., Xiong P., Li M.M.-J., Wu H. (2023). Strain Enhances the Activity of Molecular Electrocatalysts via Carbon Nanotube Supports. Nat. Catal..

[108] She F., Guo Z., Liu F., Yu Z., Chen J., Fan Y., Lei Y., Chen Y., Li H., Wei L. (2024). Curvature-Dependent Electrochemical Hydrogen Peroxide Synthesis Performance of Oxidized Carbon Nanotubes. ACS Catal..

[109] Xia H., Sun H., Yang D., Zhao J., Gao G., Wu L., Huang L., Jiang X. (2026). Curvature-Engineered Steering of Oxygen Electroreduction Pathways on Single-Atom Catalysts. Angew. Chem. Int. Ed..

[110] Zhao J., Lian J., Zhao Z., Wang X., Zhang J. (2022). A Review of In-Situ Techniques for Probing Active Sites and Mechanisms of Electrocatalytic Oxygen Reduction Reactions. Nano-Micro Lett..

[111] Wang J., Hsu C.-S., Wu T.-S., Chan T.-S., Suen N.-T., Lee J.-F., Chen H.M. (2023). In Situ X-Ray Spectroscopies beyond Conventional X-Ray Absorption Spectroscopy on Deciphering Dynamic Configuration of Electrocatalysts. Nat. Commun..

[112] Timoshenko J., Roldan Cuenya B. (2021). In Situ/Operando Electrocatalyst Characterization by X-Ray Absorption Spectroscopy. Chem. Rev..

